# Viral histones: pickpocket’s prize or primordial progenitor?

**DOI:** 10.1186/s13072-022-00454-7

**Published:** 2022-05-28

**Authors:** Paul B. Talbert, Karim-Jean Armache, Steven Henikoff

**Affiliations:** 1 Howard Hughes Medical Institute and Fred Hutchinson Cancer Center, 1100 Fairview Ave N, Seattle, WA 98109 USA; 2 Skirball Institute of Biomolecular Medicine, Department of Biochemistry and Molecular Pharmacology, New York University Grossman School of Medicine, 550 First Ave, New York, NY 10016 USA

## Abstract

The common histones H2A, H2B, H3, and H4 are the characteristic components of eukaryotic nucleosomes, which function to wrap DNA and compact the genome as well as to regulate access to DNA for transcription and replication in all eukaryotes. In the past two decades, histones have also been found to be encoded in some DNA viruses, where their functions and properties are largely unknown, though recently histones from two related viruses have been shown to form nucleosome-like structures in vitro. Viral histones can be highly similar to eukaryotic histones in primary sequence, suggesting they have been recently picked up from eukaryotic hosts, or they can be radically divergent in primary sequence and may occur as conjoined histone doublets, triplets, or quadruplets, suggesting ancient origins prior to the divergence of modern eukaryotes. Here, we review what is known of viral histones and discuss their possible origins and functions. We consider how the viral life cycle may affect their properties and histories, and reflect on the possible roles of viruses in the origin of the nucleus of modern eukaryotic cells.

**Supplementary Information:**

The online version contains supplementary material available at 10.1186/s13072-022-00454-7.

## Introduction

Eukaryotic nucleosomes, which package and regulate access to DNA, wrap 147 bp of DNA around an octameric core particle of two molecules each of the histones H2A, H2B, H3 and H4 [1]. Each of these histones has a histone fold domain (HFD) that consists of three α helices separated by two loops that together can interact with another HFD in an anti-parallel “handshake”. Despite sharing an HFD, each of the four core histones is distinct, with alignments between the different human core histones at best only on the edge of significance with E-values of 0.001 or higher. HFD proteins have a long history in all domains of life [2], but an innovation of eukaryotic histones is their ability to heterodimerize in specific pairs, H3 with H4 and H2A with H2B, that can further associate through four-helix bundles, H3 with H3’ and H4 with H2B, to form a central (H3–H4)_2_ tetramer flanked by two H2A–H2B dimers. In addition to the three-helix HFD, H3 has an additional αN helix that helps to wrap DNA, H2A has a short αN-helix and a C-terminal “docking domain” that helps to stabilize the interaction of the H2A–H2B dimers with the (H3–H4)_*2*_ tetramer, and H2B has an αC helix that together with its α3 helix forms the outer limit of the flat surface of the spool-like nucleosome. Each histone has an N-terminal and a C-terminal unstructured tail. The tails, especially the N-terminal tails of H3 and H4, have several conserved sites of post-translational modifications (PTMs) associated with gene activation or repression. A nucleosome may be further stabilized by the H1 ‘linker’ histone, which interacts with the DNA that links adjacent nucleosomes. H1 histones lack a HFD and have a separate origin from the other four histones [3], but are present in most eukaryotes [4]. A remarkable feature of the four core histones is that they are found in all eukaryotes [5, 6] spanning at least 1.6–2.4 billion years of the diversification of modern eukaryotes [7–9], and are among the most conserved proteins known. The mean amino acid identities in the HFDs to human core histones across 1208 eukaryotic genomes are 91%, 83%, 92%, and 93% for H2A, H2B, H3, and H4 [10]. Despite this strong conservation, some histones in certain protists such as H2B in *Encephalitozoon cuniculi* can have as little as 24% identity with human H2B yet are still able to form nucleosomes that bind DNA with little sequence preference [10]. In contrast to the general conservation of core histones, some histone variants, paralogs of the core histones, such as the centromere-specific H3 variant cenH3 and germline-restricted H2A variant H2A.B, have evolved rapidly and adapted for specialized functions [11, 12].

In the past two decades, homologs of the genes that encode these quintessentially eukaryotic proteins have been discovered in a growing number of double-stranded DNA virus genome sequencing projects, including in the giant viruses of the Nucleo-Cytoplasmic Large DNA Viruses (NCLDVs or *Nucleocytoviricota*). NCLDVs share genes from a core genetic content of ~ 50 genes that have been inferred to be present in a common ancestor of these viruses, though many genes have been lost or replaced in some lineages [13, 14]. Giant viruses have been variously defined by a genome size > 300 kb [15], a virion size > 200 nm [16, 17], or a proteome size of at least 500 proteins, sizes that compare to the sizes of bacteria, archaea, and picoeukaryotes [16, 18]. Their genes may encode components of the translation apparatus [19], enzymes of carbon metabolism [20], actin, myosin, and kinesins [21, 22], rhodopsins [23], and often a very large number of genes that lack homologs in other organisms (ORFans), challenging the “pickpocket paradigm” of viral genes as being derived largely or exclusively from their hosts [24], and raising the question of how often host genes are instead derived from viruses [25]. Some viruses, such as bracoviruses, have only one histone gene [26, 27], encoding a protein with high identity to the corresponding eukaryotic host histone, an apparent pickpocket’s prize from the host genome. In contrast, some NCLDVs have a complete set of all four histone genes or more, encoding highly divergent histones that appear to have anciently diverged from the corresponding eukaryotic histones prior to the diversification of modern eukaryotes [28–30], and which may be coupled in specific doublets [30–32] or even in triplets or quadruplets [20, 33]. Recently, cryo-EM structures of in vitro-assembled histone doublets from the viral family *Marseilleviridae* have been shown to form nucleosomes remarkably similar to eukaryotic nucleosomes [34, 35]. How did these histones come to be encoded in viral genomes? Do these proteins form nucleosomes in vivo? Do they interact with the host genome, or with the viral genome, or both, or neither? What are their functions? Can they be post-translationally modified like eukaryotic histones?

In this review, we collate data on the occurrence of viral histones, most of which have not been investigated, and summarize what is known about the rapidly developing field of viral histones. We discuss their possible functions, how the viral life cycle may influence their properties, whether viral replication occurs in the nucleus or cytoplasm, and discuss scenarios of their origins and evolution, particularly in the context the viral karyogenesis hypothesis of nuclear origin. Our goal is to draw attention to the perplexing diversity of viral histones and spark further investigations into their roles in viral and cellular evolution.

### Viruses that use eukaryotic histones

Histones famously package and protect DNA in eukaryotes and also have regulatory roles for processes that access DNA, such as transcription and replication. Viruses have evolved a variety of strategies to package their DNA into capsids without using histones (reviewed in Ref. [36, 37]), including even the giant mimivirus, which uses glucose-methanol-choline oxidoreductases to enclose its genome in a helical protein shell [38]. Some dsDNA viruses that do not encode histones can nevertheless utilize eukaryotic histones for their own packaging, regulation, or protection, and provide a point of reference in considering viral-encoded histones. During lytic infection of human foreskin fibroblasts, unchromatinized herpes simplex virus initially acquires histone H3-containing nucleosomes with the heterochromatic silencing PTMs H3K9me3 and H3K27me3 upon entry into the nucleus, in a defensive effort of the host cells to silence the virus. Later the viral immediate-early protein ICP0 reduces H3K9me3 on viral DNA and the immediate-early protein VP16 recruits histone acetyltransferases for H3 acetylation, a PTM associated with active chromatin [39, 40]. During latent infection in sensory neurons, in contrast, the herpesvirus proteins help to promote silencing of the episomal genome with the PTMs H3K9me3 and H3K27me3 [41, 42]. The silenced genome resides permanently in the neuron, but can occasionally re-activate to produce infectious virions.

In contrast to herpesvirus, the papillomavirus genome is packaged with host histones in the virion [43]. These histones are enriched for PTMs associated with active chromatin, which presumably reflects the chromatin state late in infection when genomes are loaded into the virions, but which may also serve to promote early transcription and replication of the viral genome upon new infection, as well as to help the viral genome evade detection by host DNA-sensing mechanisms [44]. The nucleosomes in the virion are also enriched for the replication-independent (RI) H3 variant H3.3, which in cells replaces the replication-coupled (RC) variants H3.1 and H3.2 at active sites of nucleosome turnover and is likely the major H3 variant available for packaging the papillomavirus genome during infection of non-replicating differentiated cells. These viruses demonstrate the ability to adapt to and take advantage of a eukaryotic chromatin environment for epigenetic gene regulation and virion packaging, roles that can be expanded in viruses that encode their own histones.

#### Single viral histones

A number of viruses encode a single histone that is highly similar to its eukaryotic counterpart and likely functions by being incorporated into host nucleosomes. The H4 gene of bracoviruses is the best-studied of these, but single H2Bs and H3s are also known.

### Bracovirus H4 (CvBV-H4)

**Fig. 1:**
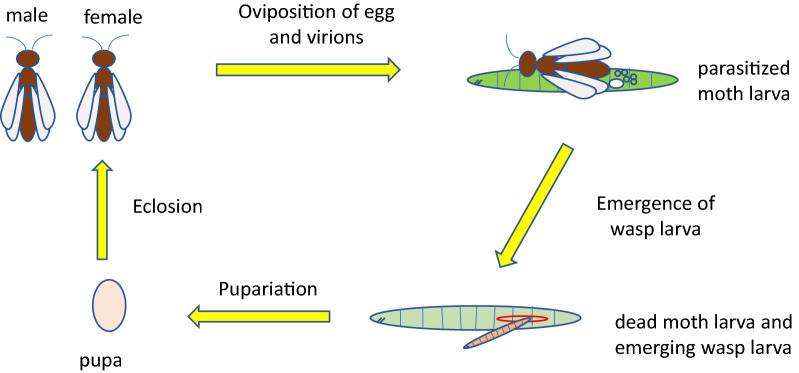
Life cycle of *Cotesia* and bracovirus. Female *Cotesia* wasps lay eggs in larvae of moths such as *Plutella xylostella* and also inject bracovirus virion particles carrying DNA circles that integrate into the chromosomes of the parasitized larvae and favor development of the wasp larvae over the moth larvae. The bracovirus provirus is resident in the wasp genome and transmitted directly to offspring

Polydnaviruses have been considered to be endosymbiont proviruses in the genomes of certain subfamilies of ichneumonid and braconid wasps, which are themselves endoparasitoids on insect larvae, especially lepidopteran larvae [45]. Female wasps of the microgastroid complex of braconid subfamilies, encompassing tens of thousands of species, lay eggs in lepidopteran host larvae and simultaneously inject virion particles (Fig. 1). Virion packaging genes are only transcribed in the calyx cells of the pupal-to-adult female ovary, where they package DNA circles amplified from the proviral segments that encode numerous virulence genes [46]. The DNA circles in the virions do not encode viral replication genes. Instead the viral genome is endogenized in the wasp genome and passed on vertically from wasp to wasp. Because the virions do not encapsidate the information for their own replication, a recent definition considers that bracoviruses and ichnoviruses are not truly viruses, but “polydnaviriformids”, though they clearly descend from viruses [47]. The viral genes in bracoviruses are derived from a beta nudivirus that integrated into an ancestral wasp some 100 million years ago [48], whereas different viruses, including an unknown member of the NCLDVs, gave rise to the ichnoviruses [49, 50].

The DNA circles in the virions integrate into the chromosomes in host cells, where expression of the virulence genes interferes with the host immune response and development, favoring the growth of the wasp larvae at the expense of the host, which is usually killed [45]. The virulence genes may be derived from the wasp, from transposable elements, or from unknown sources. One of the virulence genes in the wasp *Cotesia vestalis (*synonym *C. plutellae),* which parasitizes the larvae of the diamondback moth *Plutella xylostella,* is a viral-encoded histone H4 [26, 27]. The bracoviral H4 (CvBV-H4; accession numbers for all histones discussed in this review are found in Additional file 1: Table S1) is encoded on one of 35 genomic segments that make up the bracovirus genome of 351 kb [51, 52]. CvBV-H4 is ~ 87% identical to the H4 of *P. xylostella* and other insect H4s in the HFD and has conserved lysines corresponding to PTM sites K5, K8, K12, K16, and K20 of the eukaryotic H4 tail (Additional file 2: Fig. S1), but has an additional 38 amino acids at its N-terminus, including nine additional lysines [27]. CvBV-H4 is expressed in the nuclei of hemocytes of parasitized *P. xylostella*, which show greater H4 acetylation than unparasitized hemocytes, suggesting CvBV-H4 is acetylated. In eukaryotes, acetylation of H4 is associated with gene activity, while trimethylation of H4K20 is strongly associated with gene silencing [53]. CvBV-H4 is recovered in bulk nucleosomes from parasitized *P. xylostella* together with the endogenous H4 and other histones [54]. In vitro CvBV-H4 forms octamers with all four core histones, but not when H4 is omitted, suggesting that the octamers contain two H2As, two H2Bs, two H3s, and one each of CvBV-H4 and H4 [55]. CvBV-H4 suppresses the host immune response and delays *P. xylostella* larval development, favoring the growth of *C. vestalis*, dependent on its N-terminal tail [54, 56–58]. Chromatin immunoprecipitation of *P. xylostella* genomic sites enriched in CvBV-H4 revealed 51 sites in common between parasitized *P. xylostella* and non-parasitized *P. xylostella* transiently expressing CvBV-H4 that were not enriched in non-parasitized larvae or larvae expressing a truncated CvBV-H4 gene that lacks the N-terminal tail. Genes within 1 kb of these sites have roles in development, metabolism, immunity, signaling, and gene expression [59]. Given that H4 is deposited in insect chromatin as an H3–H4 dimer either with RC assembly of H3.2 by CAF1 or with RI assembly of H3.3 by HIRA [60], it seems likely that these 51 sites are subject to high nucleosome turnover and that CvBV-H4 is primarily deposited with H3.3 by HIRA in differentiated hemocytes and other cells. A role for the viral H4 tail in stabilizing the viral histone at specific sites is suggested by the enrichment of transiently expressed CvBV-H4, but not its truncated derivative, at these sites. Expression of CvBV-H4 results in 81 up-regulated genes, half of unknown function, and 221 down-regulated genes, 70% of which were predicted to have functions in development and metabolism [61]. Among the down-regulated genes are H4 [54], a lysine demethylase, and a SWI/SNF chromatin remodeler [62].

Virulence genes are expected to help *Cotesia* species adapt to different hosts and may therefore show signatures of positive selection. Comparison of CvBV-H4 with the viral H4s of *C. congregata* and of two incipient species, *C. sesamiae kitale* and *C. sesamiae Mombasa*, detected positive selection both in the tail and in the HFD [52]. Further examination of viral histones in 17 *C. sesamiae* populations found that polymorphisms that might reflect adaptation were primarily indels of seven amino acids in the tail (Additional file 2: Fig. S1).

The presence of bracovirus H4 genes in all investigated *Cotesia* species and their high sequence identity with insect H4s strongly suggests that an endogenous H4 gene in the ancestor of *Cotesia* species some 17 million years ago [63] was recruited to the bracovirus virulence genes to become the ancestral viral H4. The conservation of post-translational modification sites and incorporation of CvBV-H4 into host nucleosomes indicates that bracovirus H4s have been constrained to interact with the nucleosomes of the lepidopteran host species, and finding positive selection suggests that this interaction is subject to change as populations diverge. The extended tail of CvBV-H4 that suppresses the host immune response and delays larval development appears to have been weaponized by parasitic *Cotesia* species to gain an advantage against their hosts. The use of histones as weapons in microbial warfare has ample precedent [64].

### Metagenomic viral H4

While bracoviruses may have recently acquired H4 from their obligate mutualistic symbionts, more ephemeral symbiotic interactions may also lead to viral acquisition of histones. Co-infection of *Acanthamoeba polyphaga* by Marseillevirus and bacterial symbionts led to the proposal that amoebae can serve as “melting pots” for horizontal gene transfers between viruses, bacteria and their hosts [17]. Transfers can occur from host to virus or virus to host, with the latter occurring at about half the frequency of the former [25]. A marine metagenome-assembled genome (MAG) related to pandoraviruses (ERX552244.21) encodes an H4-like protein [20] that is 77% identical to human H4, and is surprisingly 93% identical to an H4-like protein encoded in a MAG [65] attributed to a Verrumicrobiales bacterium (MAD25601.1). Bacterial and archaeal MAGs sometimes contain contaminating sequences from NCLDVs. The viral H4, the bacterial H4, or both could be contaminants, but they may also potentially be recent horizontal gene transfers from the same or similar eukaryotic hosts. With only a few changed residues in the N-terminal tail and HFD and all tail lysines conserved, the H4-like protein of ERX552244.21 is likely to be incorporated into nucleosomes of its unknown host.

### *Pandoraviridae* H2Bs

The pandoraviruses have linear genomes up to 2.5 Mb [66] and are the largest of the giant viruses. Pandoravirus virions are engulfed into *Acanthamoeba castellanii* cells by phagosomes, which fuse with lysosome-like organelles that seem to stimulate the uncoating of the virions [66–68]. The capsid opens and the internal membrane fuses with the host phagosome in which they are engulfed, spilling their genome into the cytoplasm. A viral factory assembles in the cytoplasm that can recruit mitochondria and membranes, eventually leading to virion assembly and release by either exocytosis or lysis [68]. About 10% of pandoravirus genes with homologs in eukaryotes have introns, which, along with the absence of transcriptional machinery in the virion, strongly suggests that at least some viral transcription takes place in the nucleus, which disintegrates late in the infection [66]. About two thirds of genes in pandoraviruses are ORFans unique to pandoraviruses [67].

Among the minority that have eukaryotic homologs is H2B, which is present in eight completely assembled pandoravirus genomes, although homologs of H2A, H3, and H4 are absent. Pandoravirus H2Bs are 70%-75% identical to H2B from *A. castellanii* or 64–73% identical to human H2B in the HFD and in the αC helix, retaining the K120 ubiquitylation site (as numbered in human H2B), but they have modestly extended C-terminal tails, and the long N-terminal tails are divergent from eukaryotic H2Bs and from each other (Additional file 2: Fig. S2). They have ~ 15 potentially modifiable lysines in the N-terminal tail, which are similar in number though not in exact positions to the lysines in *A.castellanii* or human H2B tails. The pandoravirus H2B N-terminal tails contain 8–10 acidic residues in the first 50 amino acids while eukaryotic H2Bs lack this acidic region.

Though most pandoravirus H2Bs are ~ 200 amino acids in length, the predicted H2B protein from *Pandoravirus inopinatum* is truncated by a frameshift. In contrast, the tail of *P. celtis* has an additional 121 amino acids at the amino terminus for a total of 319 amino acids (Additional file 2: Fig. S2). A model of new ORFan gene formation in pandoraviruses proposes that new genes arise from intergenic regions that acquire transcription and translation initiation and termination signals and are then selected for function [69]. The additional 121 amino acids in the tail of *P. celtis* are consistent with such a model. Comparison of the corresponding intergenic region in the sibling virus *P. quercus*, which encodes an H2B of 198 amino acids, with the region encoding the extra 121 amino acids in *P. celtis*, reveals that the region 5′ to the *P. quercus* H2B gene has undergone a two base pair duplication in *P. celtis*. This causes a frameshift, which together with 10 single base pair substitutions including the conversion of a stop codon to a tryptophan allow an upstream methionine codon to initiate the extended *P. celtis* tail. The H2B initiation codon in *P. quercus* is still present in *P. celtis*, and it is possible that translation initiation 121 codons upstream is a misprediction, but the loss of a stop codon that permits upstream initiation may indicate that the extended N-terminus has been selected for some advantage.

The function of pandoravirus H2B is unknown. The protein is not present in the virion proteomes of five pandoraviruses [67, 70] suggesting the protein functions only in the host cell. Indeed, histone transcripts from pandoravirus relatives have been reported from virocells (infected cells reprogrammed by a virus) from the marine microbial community of the California Current [22]. The high amino acid identity of pandoravirus H2Bs with eukaryotic H2Bs in the HFD and absence of other viral-encoded histones strongly suggests that the protein assembles with host histones into nucleosomes in the nucleus, similar to CvBV-H4. Like CvBV-H4, pandoravirus H2B might suppress or re-direct the host genome to favor viral replication. The lysines in the tail may be acetylated and conservation of H2BK120 suggests that pandoravirus H2Bs can be ubiquitylated co-transcriptionally. H2BK120ub1 facilitates methylation of H3K4, H3K36, and H3K79, all of which are associated with active transcription [53]. The high identity with eukaryotic H2Bs in the HFD also suggests that this gene was acquired by an ancestral pandoravirus from a eukaryotic host and maintained under strong selection even as the pandoraviruses have diversified their genomes with new genes to the extent that the core genome common to six species represents only 15–29% of each individual genome [70].

### Single H3 histones

Several viruses encode an H3-like histone but not other histones, suggesting that these viral histones also interact with host histones to have their effects. Consistent with this, they generally have high similarity to eukaryotic H3s in the HFD (Additional file 1: Table S1, Fig. S3). Since the chaperone recognition site on α2 of H3 determines whether H3/H4 dimers can assemble into nucleosomes by the RC pathway in replicating cells and/or the RI pathway in non-replicating cells, this site is likely to be critical to the ability of the virus to interact with the host chromatin. Some but not all of these viral histones have longer N-terminal tails, similar to CvBV-H4 and pandoravirus H2Bs.

Manila clam xenomavirus is a recently described virus that may be related to papillomaviruses, polyomaviruses, and adomaviruses, which are small tumor viruses that are thought to have derived from circular Rep-encoding single-stranded DNA (CRESS) viruses [71]. Xenomavirus encodes a histone H3-like protein that is 77% identical to the H3.2 or H3.3 of Manila clam (*Ruditapes philippinarum*) in the histone fold domain and the αN helix. In the α2 helix, where the sequences SAVL (H3.2) and AAIG (H3.3) determine RC or RI assembly, respectively, xenomavirus H3 has SAIL, which would likely be permissive for assembly by either pathway if the viral N-terminal tail can substitute for the H3.2 tail, which is required for RC assembly [72]. This is questionable since the viral N-terminal tail is 35 amino acids longer than the H3.2 tail, has 17 lysines (versus 8 for H3.2), and is highly divergent with only a few scattered identities to H3.2, though these include possible homologs of lysines 18, 23, 27, 36 and 37. H3.2 and H3.3 have one difference from each other in the tail, having either A31 or S31, respectively, but the xenomavirus H3 tail has a lysine at the corresponding position of the best alignment to the H3.2/H3.3 tails. Nothing is known of the transcription, expression, or function of Manila clam xenomavirus H3.

Dishui Lake phycodnavirus (DSLPV1) is related to the *Prasinovirus* genus of the *Phycodnaviridae*, a large clade of NCLDVs that primarily infect algae [73]. The linear genome of 181 kb encodes 227 predicted ORFs, 68% of which have best blastp hits among the NCLDVs, mostly in prasinoviruses, and only four had best hits in eukaryotes. One of these four is a histone H3 that aligns to the H3.1 and H3.3 of the prasinophyte alga *Ostreococcus lucimarinus* throughout their lengths, with 87% identity and 18 amino acid substitutions. All lysines except H3K79 and most phosphorylation sites are preserved, though T31 (in H3.3) is replaced by leucine. The α2 helix chaperone recognition site has TAIL instead of SAVL (H3.1) or TAVL (H3.3), and there are a predicted 53 amino acids added to the N-terminus of DSLPV1 (Additional file 2: Fig. S3).

Single H3s are also found in marine and terrestrial MAGs. A phycodnavirus MAG (ERX552270.64) and two mimivirus-related MAGs (ERX552261.23, SRX310217.15) encode H3-like proteins [20] with 73–85% identity to H3 of the unicellular alga *Porphyridium purpureum* or *Chlorella variabilis.* PTM sites in the H3 tail are mostly unaffected except where there are deletions in two of the tails. The α2 chaperone recognition sites are partially or exactly conserved. SRX310217.15 has 68 residues added to the N-terminus including the presumed ser/thr phosphorylation hotspot TTTSSDSSSNTNRKTYQST (Additional file 2: Fig. S3). Sylvanvirus is a terrestrial MAG identified from the Harvard Forest, and may represent a new NCLDV family [74]. With a 1 Mb genome encoding 80% ORFans, it is not closely related to any known viruses, but a phylogenetic tree suggests that is most related to pandoraviruses and phycodnaviruses. Sylvanvirus encodes a histone H3 with 75% identity to human H3.1 or H3.3 throughout its length. In the α2 chaperone recognition site it encodes TAIL instead of SAVM (animal H3.1/H3.2) or AAIG (animal H3.3).

All six of these viral H3s seem likely to form nucleosomes with help from host histones. Probably all six undergo RI assembly, but only one matches H3.3 exactly in the α2 helix, and the viral chaperone recognition sequences may be adapted to assemble through either pathway. Whether any of these viral H3s can assemble into the viral genome, or whether they only work in the host chromatin is not clear. In the HFD, identity to a corresponding H3 from a possible host varies from 73 to 87%, which, together with their apparently sporadic taxonomic distribution, suggests that these are relatively recent pickpocket’s prizes. Three of these six viral H3s have extended tails and four retain most of the eukaryotic post-translational modification sites in the tails and HFD, but none retain S/T31 that characterizes eukaryotic H3.3s. The tails are diverse, with three normal-length tails, two well-conserved but extended tails, and one highly divergent tail, suggesting that the tails fulfill different functions in promoting infection in the various viral hosts.

#### Doublet histones and more

In contrast to these single viral histones, many viruses encode multiple histones, often as conjoined HFD doublets homologous to H4–H3 or H2B–H2A, which in some cases are further conjoined to other histones or non-histone proteins. These histones tend to be far more divergent in amino acid sequence than the single histones, suggesting a different evolutionary history.

### *Marseilleviridae* histone doublets

Viruses in NCLDV family *Marseilleviridae* encode distant homologs of all four core histones, arranged as joined histone doublets [17, 28, 31]. Marseillevirus T19, also known as *Marseillevirus marseillevirus*, has a genome of 368 kb encoding 457 ORFs and an icosahedral virion of ~ 250 nm, and was isolated from water in a cooling tower by co-culture with *Acanthamoeba polyphaga* [17]. Since then more than 60 marseilleviruses have been identified on five continents from water, soil, sewage, invertebrates, and humans through genomic and metagenomic sequencing [75]. Marseilleviruses have circular genomes of ~ 340–390 kb that encode 22–25 of the 26 most common NCLDV core genes and many genes specific to *Marseilleviridae* [76, 77]. A study in melbournevirus found that the capsids contain a large and dense body near the capsid internal membrane of a density suggestive of nucleoprotein complex [78]. In *Acanthamoeba ssp.*, the virions of *Marseilleviridae* are taken up by phagocytosis or endocytosis and form cytoplasmic viral factories [17, 31, 79–84]. The virions lack the viral transcription machinery and appear to rely initially on components that leak out of the host nucleus [79]. Although the host DNA remains nuclear, GFP-SUMO and other small nuclear markers leave the nucleus and spread in the cytoplasm within 30–60 min of infection, and by 2–4 h post-infection these nuclear components appear to return to the nucleus, suggesting that nuclear components necessary for viral transcription may do the same. There is no evidence that the viral DNA enters the nucleus, and in contrast to pandoraviruses, marseilleviruses lack spliceosomal introns, consistent with replication in the cytoplasm rather than the nucleus.

Phylogenetically the *Marseilleviridae* fall into five clades [84], designated A through E. *Marseilleviridae* of all five clades have two divergently transcribed genes that encode homologs of an H4–H3 doublet and an H2B–H2A doublet, and a third gene that encodes an H2A-like domain following an initial unidentified domain, though in golden marseillevirus (clade E) this latter gene appears to have undergone frameshifts in runs of Ts. For clarity, the HFDs homologous to H2A, H2B, H3, and H4 have been called Hα, Hβ, Hγ, and Hδ so that the divergently transcribed genes encode Hδ–Hγ and Hβ–Hα doublets [28]. Similarly, we will refer to the domains encoded by the third gene as Hε and Hζ, forming an Hζ–Hε doublet. The Hδ–Hγ proteins of *Marseilleviridae* are 87–100% identical within a clade, but ~ 55–65% identical between clades, and only 24% identical (E-value: 6e−10 to 1e−05) to an in silico fusion of human H4 and H3 (denoted H4 × H3, with an × inserted at the junction of human H4 NP_001029249.1 with human H3.3 NP_005315.1 for ease of visual orientation in alignments; Additional file 2: Fig. S4), except golden marseillevirus Hδ–Hγ, which does not have significant identity to H4 × H3 (E-value: > 0.05). Identity of the Hδ–Hγs to its host *Acanthamoeba* H4 and H3 is similar to that of other eukaryotes (22%, 1e−04). The Hβ–Hα proteins and the Hζ–Hε proteins are 71–100% identical within clades and 50–60% identical between clades. Hβ–Hα proteins are ~ 42% identical with an in silico fusion of human H2B and H2A (H2B×H2A, with an × separating H2B NP_066402 from H2A NP_066390.1; Additional file 2: Fig. S4), while Hζ–Hε proteins are 27–37% identical with H2B×H2A. The Hζ domain of marseillevirus T19 has been predicted to contain α helices corresponding to α1, α2, α3, and αC of H2B [35]. Hζ has few identities with human H2B, though five of those identities occur within six consecutive residues homologous to the αC helix of H2B, and Hζ also has amino acid similarities to H2B throughout the α2 and α3 helices. We therefore consider Hζ–Hε likely to be a distant homolog of H2B–H2A. This protein has also been called “miniH2B–H2A” because of its short C-terminal tail, in contrast to the long C-terminal tail of Hβ–Hα [35].

The Hδ–Hγ, Hβ–Hα and Hζ–Hε proteins are present in the virions, and Hδ–Hγ and Hβ–Hα are among the most abundant proteins, with Hζ–Hε present in lower abundance [17, 35, 78, 79], suggesting a role for these proteins in viral genome packaging. Consistent with such a role, the genes encoding these proteins are transcribed late in viral infection [85] and are located in the late-expressing “core” region of the genome with other genes encoding proteins in the virion [76]. The histones accumulate primarily in the viral factories, not the host nucleus, and are necessary for effective infection [35]. The chaperone recognition site in the α2 helix of H3.1/H3.3 is not conserved in Hγ, consistent with the notion that the viral histones are not assembled into nuclear chromatin.

**Fig. 2:**
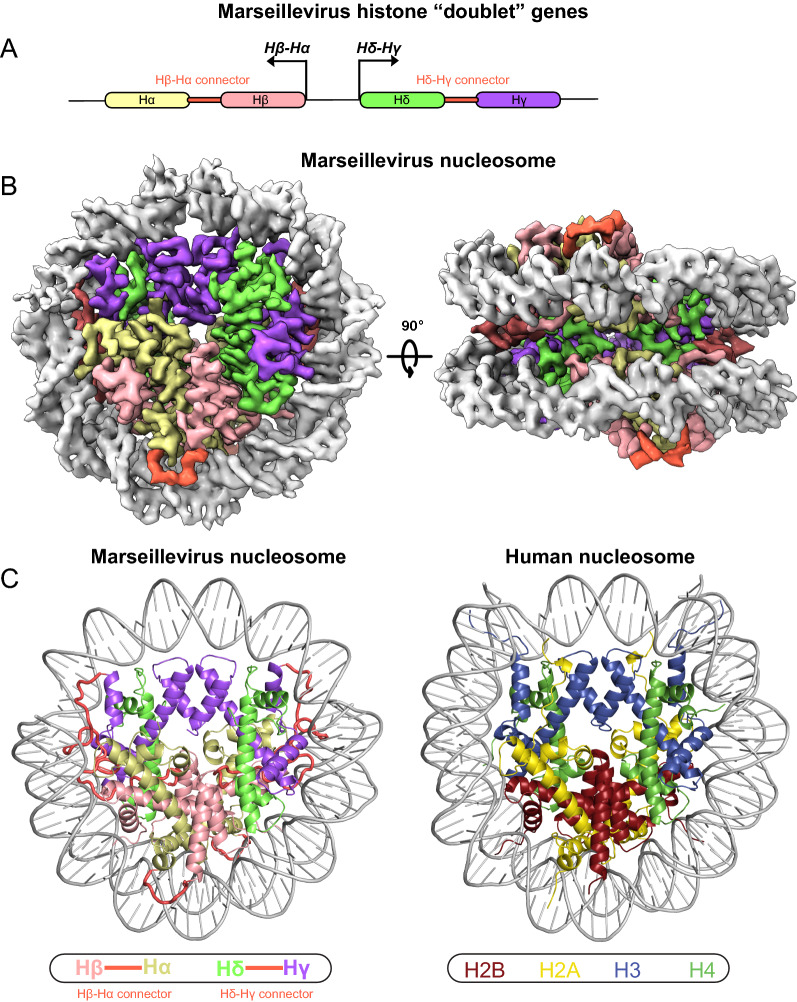
Marseillevirus nucleosome. **A** Divergent transcription of Hβ–Hα and Hδ–Hγ histone doublet genes. **B** Cryo-EM structure of Marseillevirus nucleosome made up of a tetramer of two Hβ–Hα and two Hδ–Hγ proteins. The regions linking the separate histone fold domains are marked in red. **C** Comparison of the Marseillevirus nucleosome and a human nucleosome. Reprinted from Ref. [34], used with permission

Cryo-EM structures of Hδ–Hγ and Hβ–Hα from marseillevirus T19 or from melbournevirus assembled onto the Widom 601 histone positioning sequence (Fig. 2) reveal that the histone proteins from each of these viruses can assemble into a structure remarkably like the eukaryotic nucleosome [34, 35]. The Hβ–Hα proteins are identical in these two viruses, while the Hδ–Hγ proteins differ by a single residue. In the viral nucleosomes, the two domains in each doublet fold with each other, then they assemble into a tetramer by four-helix bundles between Hγ and Hγ’, and between Hδ and Hβ, equivalent to the way eukaryotic histones assemble into octamers. Nucleosome core particle assembly is mostly mediated by hydrophobic and a few electrostatic interactions, although the precise residues involved are only partially conserved with eukaryotic histones. The intramolecular R to D salt bridges stabilizing α2 and α3 in H3 and H4 are conserved in Hδ–Hγ. Notably, the αN helix, which in H3 stabilizes the DNA at its entry and exit points, is shorter in Hγ, contributing to the viral tetramers organizing only  ~ 121 bp in the cryo-EM structures, in contrast to eukaryotic nucleosomes that wrap 147 bp of DNA [34, 35]. An RQ motif in the Hδ–Hγ connector contacts the DNA [34]. Arginines that interact with DNA via in the minor groove are conserved in Hα. Arginines in Loops 1 and 2 of H4 and H3 that contact the minor groove of DNA are not conserved. The H4R45–H3T118 pair that reaches into the minor groove in eukaryotes is swapped with an HδS43–HγR199 pair in the minor groove in the viral nucleosome, and H3R83 is replaced by HγH164. The acidic patch of eukaryotic nucleosomes that is formed from eight negatively charged residues of H2A and H2B, which interacts with chromatin remodelers and the H4 tail of adjacent nucleosomes, is partially conserved in the viral nucleosomes, with two fewer acidic residues [34, 35]. It is possible that the acidic patch can interact with other viral chromatin proteins, or that it interacts with the positively charged Hδ tail, although the tail appears to fold back to interact with its own α2 and α3 helices.

The N-terminal tails of H3 and H2A are substituted by shorter connector sequences between the HFDs in Hδ–Hγ and Hβ–Hα, while the N-terminal tail of Hβ is ~ 20 residues shorter than that of eukaryotic H2Bs and the C-terminal tail of Hα is ~ 50 residues longer than that of H2As. While lysines and serines that are modified in the eukaryotic histone tails are mostly not conserved in *Marseilleviridae* histones, there are four lysines and two serines in the N-terminal tail of Hδ–Hγ and two lysines and five serines in the connector, three lysines and one serine in the Hβ N-terminal tail, and 23 lysines and 9 serines in the Hα C-terminal tail, raising the possibility that the viral histones may be modified. For example, rapid acetylation of the Hα C-terminal tail upon infection might facilitate histone removal for viral transcription and replication.

To assess whether viral nucleosomes assemble on marseillevirus DNA in virions, capsids from marseillevirus T19 and a more recent isolate marseillevirus G648 were opened and permeabilized [86]. The chromatin was digested with micrococcal nuclease (MNase), revealing densely packed ~ 121 bp particles resistant to internal cleavage and lacking linker DNA, with dinucleosome and trinucleosome sizes of ~ 270 and ~ 390 bp, in contrast to *Drosophila* nucleosomes with mono-, di-, and trinucleosome sizes of 150, 330, and 540 bp. These viral nucleosomes were not phased over genes, as is typical in eukaryotes. Cleavage with methidiumpropyl-EDTA-Fe(II), or MPE [87], gave similar mono- and dinucleosome size fragments for *Drosophila*, but gave a broad distribution of fragment sizes with a peak at 147 bp for marseillevirus chromatin, and no di- or tri-nucleosome peaks, suggesting packaging is too tight for MPE to cleave effectively between nucleosomes [86]. The endonucleolytic activity of MPE may generate random cleavages and then trim them to the most common size of 147 bp DNA that can be protected by the marseillevirus nucleosome, while the exo-endonuclease activity of MNase more frequently cuts between nucleosomes and nibbles loose ends to the minimally protected 121 bp particle. When assembled on tandem arrays of the Widom 601 nucleosome positioning sequence, 39% of MPE fragments were precisely positioned as mono-, di- or trinucleosome cleavages. In contrast 73% of control *Xenopus* nucleosomes were precisely positioned. MNase digestion gave a distribution of discrete fragments 20–25 bp smaller than MPE, as was observed in virio, though these precisely cut fragments made up only 1% of the total. These observations strongly suggest that marseillevirus nucleosomes have evolved to maximally pack viral DNA and can largely overcome positioning cues.

A role in packaging does not necessarily preclude other roles, such as protecting the DNA from host mechanisms that sense or restrict invading viral DNA. Although the dense packing and lack of genic phasing argue against a role in gene regulation, it is possible that the Hζ–Hε doublet could have a role in initiating transcription by replacing Hβ–Hα, similar to the H2A.Z variants of eukaryotes that replace H2A [86]. Heterotrimeric nucleosome-like structures lacking one Hβ–Hα unit were also recovered in cryo-EM, suggesting a possible diversity of nucleosome-like particles [34].

To test whether the Hα, Hβ, Hγ, and Hδ single HFD moieties from *Marseilleviridae* clades A, B, C, and D show specific relationship to any of six eukaryotic lineages (Metazoa, Fungi, Amoebozoa, Archaeplastida, Heterolobosea, and Euglenozoa) or to their histone variants such as cenH3 or H2A.Z, which could suggest a “late” horizontal gene transfer, the moieties were separated in silico and a phylogenetic tree was constructed which confirmed the specific relationships of the Hα, Hβ, Hγ, and Hδ moieties to H2A, H2B, H3, and H4, respectively, to the exclusion of archaeal histones [28, 34]. All four viral moieties from the four clades of *Marseilleviridae* branched as sisters of the corresponding eukaryotic histones, prior to the divergence of modern eukaryotic histones and their variants. This suggests common ancestors for the corresponding viral and eukaryotic moieties at an “early” proto-eukaryotic stage of evolution, a hypothesis supported by the large *Marseilleviridae* topoisomerase II proteins of nearly 1200 amino acids, which similarly branch as sister to all eukaryotic topoisomerase II proteins to the exclusion of the homologous archaeal gyrases [28]. This branching is also consistent with several studies finding that the origin of NCLDVs predates the divergence of modern eukaryotes [13, 21, 88, 89], and with the high conservation rate of NCLDV core genes in *Marseilleviridae* [76, 77]. In either the “early” or “late” scenarios, divergence from eukaryotic or proto-eukaryotic histones may have been driven by the need to distinguish viral histones from host histones in cellular location, properties, and functions. Unlike the single viral histones we previously discussed, *Marseilleviridae* histones do not appear to interact with host histones [35], nor do they appear likely to interact with most host chaperones and modifiers, although the lysine-rich Hα tail strongly suggests some modification.

### Insect iridovirus histone doublets

**Fig. 3:**
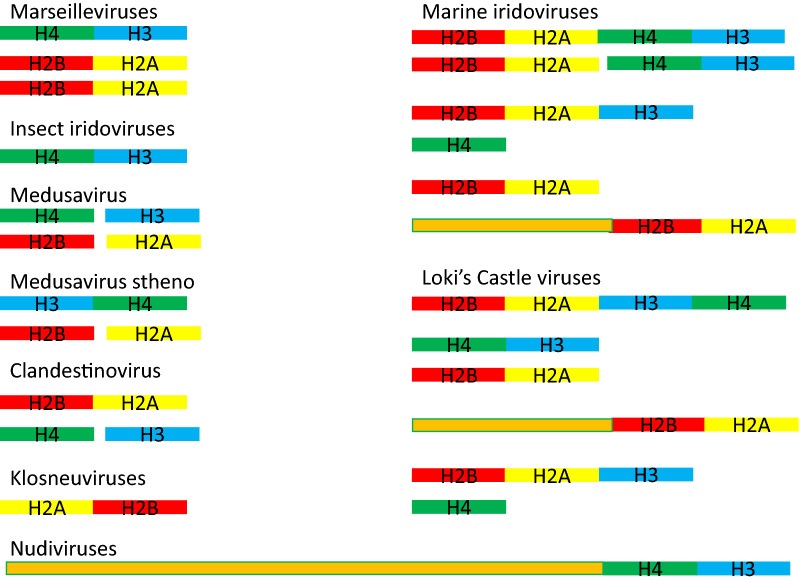
Viral histone doublets, triplets, and quadruplets. Configurations of predicted histone fold domain proteins in various viruses and metagenomic viral genomes. Green: H4-like; blue: H3-like; red: H2B-like; yellow: H2A-like; orange: no homology to any known protein

Invertebrate iridoviruses (or iridescent viruses) are viruses of insects and crustaceans. They include the ascoviruses and are relatives of marseilleviruses [90]. Iridoviruses get their name because infected hosts may have an iridescent discoloration of the cuticle. Iridoviruses have linear, circularly permuted genomes with redundant ends [91], and replicate initially in the host nucleus before exporting their genomes to the cytoplasm for further replication and packaging [92]. An H4–H3-like doublet has been found in five invertebrate iridoviruses: IIV9 from the moth *Wiseana spp.*, IIV22 and IIV25 from the black fly *Simulium spp.*, IIV30 from the corn earworm moth *Helicoverpa zea*, and the mosquito *Anopheles minimus* iridovirus. These doublet proteins are 87–100% identical to each other, except *Anopheles minimus* iridovirus, which is only about 53% identical to the others (Additional file 2: Fig. S5; Fig. 3). All are approximately 23% identical to H4xH3, and are not significantly similar to marseillevirus Hδ–Hγ. As with Hδ and Hγ, the H4-like and H3-like HFDs branch in a phylogenetic tree prior to the divergence of eukaryotic H4s and H3s, respectively [29]. No homologs of H2A or H2B were found in any iridovirus genome using human H2A, human H2B, marseillevirus Hβ–Hα and marseillevirus Hζ–Hε as queries for tblastn searches.

It is unknown whether the insect iridovirus doublet histones bind to DNA. The arginine in eukaryotic H4 that binds the minor groove of DNA is conserved in the iridovirus doublet, but the three arginines in H3 that bind in the minor groove are not, though possibly nearby arginines or lysines could substitute (Additional file 2: Fig. S5). The protein is present in both the virion and in infected cells [93], suggesting a role in packaging DNA. Whether it forms (H4–H3)_2_ dimers or higher multimers, or joins with host H2B and H2A to form core particles similar to histone octamers is unknown. Since replication happens initially in the host nucleus, it seems plausible that the doublet could interact with host H2A, H2B, and DNA as well as with viral DNA. However, the sequence at the H3 α2 chaperone recognition site of the doublet is highly divergent, raising doubts as to how it would become assembled into host chromatin. It also lacks the post-translationally modified tails of H4 and H3 and has no lysine-rich tail like some other viral histones, so it probably is not modified, or minimally so. These features suggest that it may not function in the nucleus but only later when iridovirus replication moves to the cytoplasm for virion packaging.

### Medusavirus and clandestinovirus histones

Medusavirus represents a distinct family of NCLDVs in the order *Pandoravirales* [94] with a linear genome of 381 kb and an icosahedral capsid of approximately 260 nm with an internal membrane enclosing the DNA inside the capsid [29]. In culture, the virion is taken up by *Acanthamoeba castellanii* by phagocytosis, and once in the cytoplasm fuses with the nucleus where the virus replicates, in contrast to the cytoplasmic replication of *Marseilleviridae*. Consistent with nuclear replication, the genome does not encode a DNA-directed RNA polymerase, a topoisomerase, or an mRNA capping enzyme, all of which are typically found in NCLDVs [13], suggesting medusavirus is dependent on the host nucleus for these functions. The genome contains introns that are presumably spliced out in the host nucleus [29]. A viral factory forms in the nucleus and has not been observed in the cytoplasm [95]. *Acanthamoeba castellanii* may be a natural host for medusavirus as there is evidence of extensive horizontal gene transfer between medusavirus and *Acanthamoeba* in both directions, including an H1-like protein encoded in the virus that appears to be acquired from the host, though virus to host transfer is also possible.

Homologs of all four core eukaryotic histones are encoded separately by medusavirus (Fig. 3), and the proteins are found in the virion, suggesting a packaging function [29]. A second medusavirus, called medusavirus stheno, also encodes all four core HFDs, but in this case the H3-like and H4-like HFDs are encoded as an H3–H4 doublet [32]. The core histones are transcribed at an intermediate time during infection, but the H1-like protein is expressed early, suggesting that it acts independently of the core histones [95], perhaps to shut down the host genome. Like the marseillevirus HFDs, all of the medusavirus HFDs branch below the divergence of the corresponding eukaryotic histones in a phylogenetic tree [29].

The extremely divergent H4-like HFD in medusavirus is identified by homology to the H4 superfamily in the Conserved Domain Database (E value: 4e−03), but a blastp search using it as query picks up only the H4-like HFD of the H3–H4 doublet of medusavirus stheno and no eukaryotic H4s (E value: > 0.05), including *Acanthamoeba* H4 (Additional file 2: Fig. S6). In stark contrast, the H3-like HFD of medusavirus is 44% identical to *Acanthamoeba* H3 and other eukaryotic H3s, and most tail lysines are conserved with eukaryotic H3s (except H3K4). The α2 chaperone recognition sites in the medusaviruses have partial identity to that of *Acanthamoeba*, suggesting the possibility that they may be recognized by host chaperones. The H2B-like HFDs of medusavirus and medusavirus stheno differ from *Acanthamoeba* H2B in having longer N- and C-terminal tails and a longer Loop 1. Some tail lysines are conserved with *Acanthamoeba*, including the K120 ubiquitylation site, but there are seven additional lysines in the C-terminal tails, and the N-terminal tails have 11 additional acidic residues. The H2A-like HFDs of medusavirus and medusavirus stheno also have extended, more acidic N-terminal tails than *Acanthamoeba* H2A.X and lysine-rich C-terminal tails of ~ 90 residues.

The medusavirus relative clandestinovirus was recently cultured in *Vermamoeba vermiformis* [30]. The linear 582 kb genome similarly encodes HFDs corresponding to all four core eukaryotic histones. While H3 and H4 homologs are separately encoded as in medusavirus, H2A and H2B homologs are encoded as an H2B–H2A-like doublet (Fig. 3). Clandestinovirus also encodes an H1-like protein with a WWE-domain, a protein interaction domain that is associated with enzymes in ubiquitin and ADP-ribose conjugation systems [96] and several other proteins in amoebae, raising doubts as to whether this H1-domain-containing protein functions in condensing chromatin. Like medusavirus, clandestinovirus enters the host by phagocytosis and fuses with the nucleus, which it turns into a viral factory for replication. Also like medusaviruses, clandestinovirus has introns in some genes that are presumably spliced out in the host nucleus.

The clandestinovirus H4-like histone is 30% identical to *Acanthamoeba* H4 in the HFD. Identities in the N-terminal tail are minimal, but may include three lysines (Additional file 2: Fig. S6). The clandestinovirus H3-like HFD is 43% identical to *Acanthamoeba* H3 and is identical in the α2 chaperone recognition sequence. It has a longer Loop 1 and a shorter N-terminal tail, which nevertheless has five or more potentially modifiable conserved lysines [30]. The H2B-like moiety of clandestinovirus H2B–H2A is only 25% identical to human or *Acanthamoeba* H2B over most of its length and up to five lysines may be conserved in the N-terminal tail together with H2BK120 in the C-terminal tail. The H2A-like moiety of clandestinovirus H2B–H2A is 33% identical to eukaryotic H2As in the HFD and the C-terminal tail has an additional 54 residues with 15 lysines and 12 acidic residues.

The medusaviruses and clandestinovirus have more conserved residues that are modification sites in the corresponding eukaryotic histones than the marseilleviruses or iridoviruses, though surprisingly, the H4-like protein of medusavirus has no significant similarity to H4 while the H3-like protein is 44% identical to H3. The difference in the divergence of H3-like and H4-like histones in medusavirus from eukaryotic histones might have a number of explanations, but one possibility is that the H3-like member of an older more divergent H4–H3-like pair, possibly present in a doublet, was replaced by a younger H3 or H3-like histone borrowed from its host at some point in the past, perhaps conferring some advantage for replication within the nucleus. Such replacement of a member of an ancestral pair might be the cause of forming the singlets and reversed H3–H4 doublet present in the medusaviruses and in clandestinovirus. Whether or not this scenario is correct, the presence of similar chaperone recognition sites, conserved modification sites, and extended lysine-rich tails suggests that these histones interact with host DNA, chaperones, and histone modifying enzymes. This contrasts with the insect iridoviruses, which also replicate at least partially in the nucleus, but their H4–H3-like histones do not show the same conservation of modification sites and the chaperone recognition site.

### Nudivirus Orf1

The first sequenced viral histone-like gene was not from an NCLDV but from the corn earworm moth nudivirus, *Helicoverpa zea* (formerly *Helothis zea*) nudivirus 1, or HzNV-1 [97] and its 93.5% identical variant HzNV-2 [98]. Nudiviruses are viruses of arthropods related to baculoviruses, with circular genomes of 100–230 kb that replicate in the host nucleus [99]. HzNV-2 virions accumulate in the reproductive tissues of both male and female moths, are sexually transmitted, and can pass virions vertically from females to their offspring. Integration of a similar virus into the genome of the ancestor of braconid wasps presumably gave rise to the bracoviruses [48]. Orf1 of HzNV-1 and HzNV-2 encodes a protein of 1111 residues, of which the final 200 are homologous to an H4–H3-like doublet with 30–40% identity to the HFDs of insect H4 and H3. The protein shows no homology to other known proteins in its first two thirds and has a middle region rich in serines and prolines. Its function is unknown, and the role of the HFDs is obscure. Possibly it could serve as a host poison, binding up chromatin interacting proteins. While it seems highly unlikely that it could function to wrap DNA, large domains and long tails are known in some eukaryotic histones, such as the 240 residue macro domain fused to H2A in the macroH2As of animals, and the long tails on cenH3s in many eukaryotes (e.g., the 170 residue cenH3 tail of the red alga *Cyanidioschyzon merolae* and the > 520 residue cenH3 tail of the cellular slime mold *Dictyostelium discoideum*).

### Histone diversity from NCLDV MAGs

Metagenomics projects have yielded a wealth of new NCLDV genome sequences with a surprising diversity of histones (Additional file 1: Table S1). These genomes encode histone singlets, doublets, triplets, and quadruplets in a variety of combinations, as well as histones fused to proteins of unknown function (Fig. 3).

Klosneuviruses, including indivirus and klosneuvirus KNV1, are related MAGs of 0.86 and 1.57 Mb, respectively, and form a distinct NCLDV clade within the mimiviruses [19, 94]. Their hosts are believed to include cercozoan protists and ciliates [19]. Indivirus and klosneuvirus KNV1 encode distantly related HA–H2B-like doublet histones, reversed in order from the more common H2B–H2A-like doublets (Additional file 2: Fig. S7). The doublets lack homology to the N-terminal and C-terminal tails of both H2A and H2B, and are 8 to 13 residues shorter in the region corresponding to H2B α2-Loop2-α3. No homologs of H3 and H4 were detected in tblastn searches of these viral genomes. Whether these apparently shortened H2A–H2B-like doublets can properly fold into an H2A–H2B-like dimer that wraps viral or host DNA is unclear, as is whether they interact with host H3 and H4. One possibility is that they may act to destabilize host nucleosomes, assembled on either the viral or host genome.

Marine iridoviruses are known only from MAGs and form a separate clade from the invertebrate iridoviruses described above and from vertebrate iridoviruses that lack any known histones [20], but they encode a variety of histones (Fig. 3), all of which lack homology to the tails of eukaryotic histones (Additional file 2: Fig. S8). The marine iridovirus MAG SRX802077.164 encodes two full sets of histones: an H4–H3-like doublet, an H2B–H2A-like doublet, an H2B–H2A-H3-like triplet and an H4-like singlet. Three related MAGs (SRX802202.41, SRX802963.105, and SRX802143.125) also each encode H2B–H2A–H3-like triplets and singlet H4-like proteins. A fourth MAG ERX552261.56 encodes a related H4-like protein, but no corresponding H2B–H2A–H3-like protein was recovered. SRX802076.27 and SRX803008.97 encode H2B–H2A-like doublets, and SRX802202.32 encodes a protein in which an H2B–H2A-like doublet is fused downstream of 200 amino acids with no known homology.

Metagenomic sequences from sediments of the Loki’s Castle hydrothermal vent field on the mid-Atlantic Ridge [33] yielded four genomes related to marseilleviruses (LCMAC101, LCMAC102, LCMAC201, LCMAC202) and 3 related to pithoviruses (LCPAC001, LCPAC102, and LCPAC304) that encode histone-like proteins (Additional file 2: Fig. S9). In a phylogenetic tree, all of these seven histone-encoding genomes form early branches prior to the divergence of their previously known marseillevirus or pithovirus relatives [33]. As with marine iridoviruses, histone singlets, doublets, triplets, and quadruplets are found in Loki’s Castle genomes in perplexing combinations (Fig. 3). The viral genome LCMAC101 encodes an H4–H3-like doublet, an H2B–H2A-like doublet, and another protein that begins with an H2B–H2A-like doublet, but this is fused to another ~ 190 residues that are related to an adjacent LCMAC101 gene encoding a hypothetical protein (QBK85836.1). LCMAC101 and LCMAC102 both encode H2B–H2A–H3–H4 quadruplets. Like marine iridoviruses, LCMAC102 also encodes an H2B–H2A–H3-like triplet together with an H4-like singlet. LCMAC201, LCMAC202, and the pithovirus-related genome LCPAC001 encode H2B–H2A-like doublets, while LCPAC304 encodes two different H4–H3-like doublets, two H2B–H2A-like doublets, and a singlet H4-like protein with a 100 residue N-terminal tail. LCPAC304 additionally encodes a protein with an H1-WWE-like protein, which is the closest known homolog to the H1-WWE protein of clandestinovirus, with similar caveats as to its role in chromatin. An H1-like domain is also found in the pithovirus LCPAC102, but it encompasses only 76 residues of a 330 residue protein, and the sole marginally significant blastp hit is to an H1 from the cellular slime mold *Heterostelium album* (E value: 0.041). With several of these genomes encoding multiple sets of histones, they might form nucleosomes or nucleosome-like structures from different combinations of doublets, quadruplets, singlets and triplets, or they might even form hemisomes [100] from a single quadruplet. It seems likely that in genomes with multiple sets of histones, the different sets may be specialized for different functions such as packaging or transcription.

*Marseilleviridae, Pithoviridae*, and *Iridovirida*e are major families in the NCLDV order *Pimascovirales*, but histone fusions are also found in MAGs of the order *Imitervirales* encompassing the *Mimiviridae* and their numerous relatives. The mimivirus-related MAG SRX802963.76 encodes both an H2B–H2A-like doublet and an H4–H3-like doublet. ERX552270.89 encodes an H2B–H2A–H4–H3-like quadruplet and an H2A-like singlet, and ERX552261.3 encodes an identical H2A-like protein. ERX556315.47 and ERX552270.68 encode 200 and 312 residue H2A-like proteins which include regions that HHpred suggests could be homologous to H2B. Two ‘late phycodnavirus’ [20] MAGs (ERX555907.31 and ERX555967.39) encode H3-like proteins with N-terminal extensions of approximately 170 residues homologous to no known proteins.

**Fig. 4:**
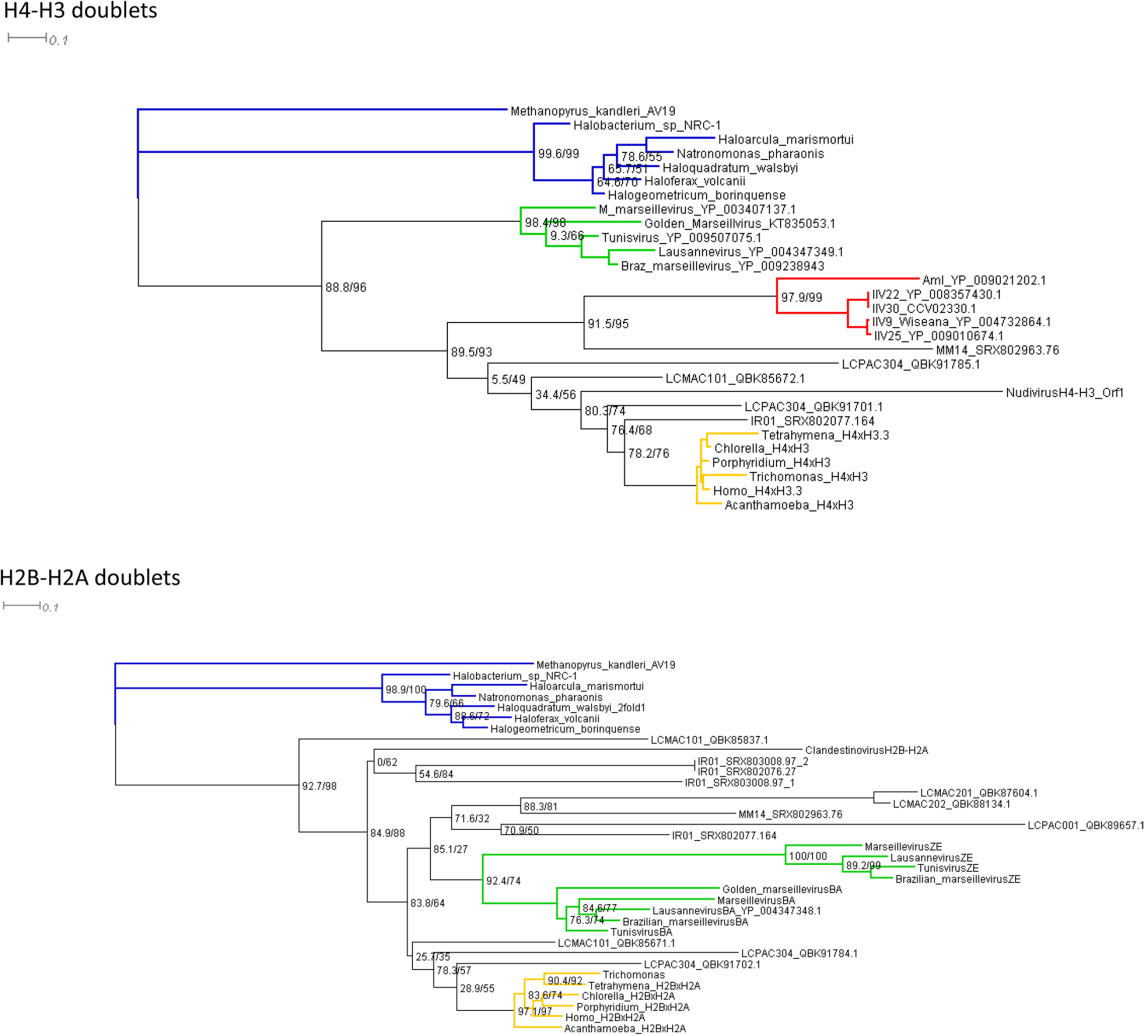
Maximum Likelihood trees of histone doublets. The trees are unrooted, but drawn as if rooted on archaeal doublets. Archaea (blue); eukaryotes (orange); *Marseilleviridae* (green); insect iridoviruses (red). Alignments were made with EMBL-EBI Muscle software and manually trimmed to remove the N-terminal tails, leaving the histone fold domains, the αN helix and docking domain of H2A, and the αC helix of H2B. The alignments were used to make maximum likelihood trees with IQ Tree [129] and viewed and annotated with Dendroscope 3.0 [130]. Numbers in parentheses are SH-aLRT support (%)/Ultrafast Bootstrap support (%)

For the most part, none of these histones from MAGs show high primary sequence identity either to the corresponding eukaryotic histones or to each other (Fig. 4). These genomes indicate a considerable diversity in encoded histones in terms of primary sequence, number of HFDs, and even the order of HFDs in doublets and quadruplets. This suggests a long and complicated evolutionary trajectory for these histones.

### *Origins?* The significance of doublets

Viruses encoding histones fall into two clearly distinguishable classes: those genomes encoding a single histone and those genomes encoding two or more histones, predominantly as H4–H3 or H2B–H2A doublets. The single histones are mostly 73–89% identical to their eukaryotic counterparts in the HFD. The conservation of eukaryotic PTM sites in viral tails varies, but frequently there are identities. CvBV-H4 is known to be incorporated into *Plutella* chromatin, and the six singlet H3-like histones all appear likely to be incorporated into host chromatin based on similar chaperone recognition sites. Such singlet histones are found scattered among at least five NCLDV families as well as in the unrelated xenomavirus and bracovirus. The high conservation of the HFDs suggests a “recent” origin from a eukaryotic host from which the gene has been pickpocketed [24]. In the only datable case of viral histone acquisition, “recent” acquisition for bracovirus CvBV-H4 means an estimated 17 million years ago.

In contrast, the histone doublets encoded by viruses range from 22 to 48% identity in the HFDs, with medusavirus H4 an outlier showing no significant identity to eukaryotic H4, along with the possible H2B domains in the large H2A-like proteins of some marine mimivirus-like MAGS. Except for nudivirus Orf1, these doublets are all found in NCLDVs, including *Marseilleviridae, Iridoviridae, Pithoviridae, Mimiviridae* and relatives, and the medusaviruses. The configuration of HFDs predominantly in doublets, with some triplets, quadruplets, and singlets, is a significant common feature. While it remains possible that the lesser identity of these HFDs with eukaryotic histones might reflect an accelerated evolution of more “recently” acquired eukaryotic histones, rather than a more ancient proto-eukaryotic origin as is suggested by phylogenetic trees, the preponderance of doublets is surprising, since we might expect single histones to be the most common class or the only class, as it is with those single histones with 73–89% identity to eukaryotic HFDs.

The occurrence of histone doublets is interesting in the context of a potential common ancestor with proto-eukaryotic histones because doublets are found in some archaeal histones of the HMfB family [101, 102] and in a class of HFD proteins found in bacteria and some archaea [2, 102]. It has been proposed that the occurrence of histone doublets was an intermediate stage in the evolution of eukaryotic histones that forced the formation HFDs that heterodimerize with their specific HFD partners as in eukaryotes, rather than form homodimers or random assortments of HFD paralogs, as in most archaeal histones of the HMfB family [103]. No fused doublets, triplets or quadruplets have been confirmed among eukaryotic histones, though blastp searches using fused viral histones as query yield many such putative fusions among animals, which cluster their histone genes. In cases that we have investigated (e.g., alligator gar *Atractosteus spatula* putative H2B–H2A, Brandt’s bat *Myotis brandtii* putative H4–H3), these putative fusions appear to be mis-annotations of splicing patterns, where nearby histones have been mispredicted to be spliced to each other, even though the expected start and stop codons of the single histones are present in the appropriate positions. However, we cannot rigorously exclude the possibility that some of these predicted eukaryotic histone fusions may be real. Nevertheless, the preponderance of doublets in NCLDVs whose ancestor originated prior to the divergence of modern eukaryotes suggests that doublet histones may have been usual at the time(s) these viral histones diverged from their eukaryotic counterparts.

The presence of highly divergent H4–H3-like and H2B–H2A-like doublets in the *Pimascovirales* [94] encompassing the *Marseilleviridae, Iridoviridae*, and *Pithoviridae*, and also in the *Pandoravirales* (medusavirus) and *Imitervirales* (mimivirus relatives), suggests that these proteins are vertically descended from a common viral ancestor rather than being independently captured from a proto-eukaryote. However, neighbor-joining trees present a more complicated picture (Fig. 4). While the histones of cultured viruses group with their relatives in a tree, with all of the Hβ–Hαs together, the Hζ–Hεs together, the Hδ–Hγs together, and the insect iridovirus H4–H3-like histones together, the doublets from MAGs are mostly widely dispersed from their presumed nearest relatives. For example, the H2B–H2A-like doublets of LCMAC marseillevirus-like viruses, including two from the same genome, appear on three widely separated branches. This might indicate that LCMAC H2B–H2A-like histones are significantly older or more rapidly evolving than marseillevirus histones, or that some LCMAC H2B–H2As have been replaced by other highly divergent H2B–H2A-like doublets from proto-eukaryotes or their viruses, or it might indicate that the MAGs are artificial chimeric genomes with histones derived from different genomes than are the handful of core genes used to assign each MAG into a taxon. Further investigation and culturing of MAGs are needed to resolve these alternatives. What is clear is that doublets are highly diverse and widespread. It is possible that histone doublets were ancestral in all NCLDVs, but frequently lost in some lineages, or that they were limited to the class *Megaviricetes*, excluding the poxviruses and asfarviruses (class *Pokkesviricetes*) in which no histones have been reported. The reversed doublets and the singlets of klosneuviruses and medusaviruses may represent independent acquisitions from eukaryotes or from distinct proto-eukaryotic lineages, or they may represent the disjoining and rejoining or replacement of ancestral HFD doublets. Whether doublets or singlets were ancestral in *Megaviricetes*, the occurrence of triplets, quadruplets, and reversed doublets indicates some selective pressure for fused histones, probably to ensure coordinate and stoichiometric expression of histone HFDs that have distinct but coordinated functions. Similar fusions of proteins of related function have been observed in NCLDV glycolytic enzymes [20] and between D5R helicases and primases [13]. Such a selective pressure to fuse HFDs that have evolved distinct functions is still compatible with the notion that doublets may have facilitated the original differentiation of those functions by constraining HFDs in forced pairs.

With the exception of medusavirus, clandestinovirus, and the “recent” single histones, viral histone tails are not usually obviously homologous with the corresponding eukaryotic tails, and the chaperone recognition site on H3-like proteins is not conserved. The absence of chaperone recognition sites and of extended tails with eukaryotic PTM sites in most doublet histones is reminiscent of archaeal histones and, together with HFD phylogenetic trees, also suggests derivation of these histones prior to modern eukaryotes. The lack of tails in most archaeal histones is a distinguishing feature compared with eukaryotic and many viral histones. The extension of the *P. celtis* H2B N-terminal tail presents a possible model for the origin of eukaryotic histone tails. Archaeal histones assemble on DNA and permit transcription and replication without any known chaperones or remodelers (reviewed in Ref. [104]). Whereas archaeal histones may oligomerize as homo- or hetero-dimers through forming four helix bundles between 3 and 5 or more dimers into extended and somewhat flexible “archaeasomes” that seem not to be separated by linkers, histone doublets in the archaea *Methanopyrus kandleri* and *Haloferix volcanii* form obligate heterodimers between the diverged N-terminal and C-terminal HFDs of the doublet, and these doublets dimerize and appear to form tetra-HFD “nucleosomes” of a fixed size [101]. Whether viral histone doublets assemble without host chaperones or remodelers and whether they form discrete nucleosomes in vivo remain to be investigated.

### The viral karyogenesis hypothesis

In invoking a possible common ancestor of doublet viral histones and those of proto-eukaryotes, it is helpful to clarify the term proto-eukaryote. Cavalier-Smith distinguished between a proto-eukaryote cell with a nuclear envelope and cilium and a prekaryote cell lacking these features, which might nevertheless have other eukaryote-specific properties [105]. Here we use proto-eukaryote in the more general sense of any cell on the evolutionary trajectory from the first eukaryotic common ancestor (FECA) to the last eukaryotic common ancestor (LECA). No proto-eukaryotes exist today, having been presumably competitively eliminated by the highly sophisticated and more successful LECA and its descendants [106], so we can only attempt to infer the many steps along their journey. We use the term prekaryote in a similar sense to Cavalier-Smith to indicate a proto-eukaryotic cell that has not yet evolved a nucleus. It is thus the same as the term prokaryote except that prekaryote refers specifically to a proto-eukaryote, whereas prokaryote by convention is generally understood to mean bacteria and archaea.

The discovery of giant viruses has brought reconsideration of the nature of viruses and their role in eukaryotic evolution [24, 107, 108], including re-considerations of the viral eukaryogenesis hypothesis [109–111], in which a virus in a prekaryote played an instrumental role in the origin of the nucleus. Here we prefer the term “viral karyogenesis”, for this hypothesis of nuclear origin, since modern eukaryotes are defined by a large suite of unique features in addition to their nuclei, at least some of which (e.g., mitochondria) are not of viral origin [106]. Since histones are essential features of eukaryotic nuclei, we here briefly consider this hypothesis in the context of viral histones.

In 2000 Villarreal and DeFilippis proposed that Pol δ of the B Family of DNA polymerases, primarily responsible for lagging strand synthesis during eukaryotic replication, was derived from a virus similar to phycodnaviruses [112]. Shortly afterward, Takemura proposed that Pol α, which initiates synthesis from the RNA primer, was derived from a poxvirus, and noting the complex genome, linear chromosome, and replication in the cytoplasm, he proposed that the nucleus derived from an engulfed orthopoxvirus that formed a symbiotic relationship with an infected archaeal cell [113]. He later updated the hypothesis by proposing that the B family polymerases diversified in NCLDVs and that α, δ and ζ polymerases were all acquired from different viruses at different stages of proto-eukaryotic evolution [89]. Endogenized NCLDV genomes, which are common in green algae [114] and in other unicellular aquatic eukaryotes [115], may be up to 1925 kb and encode up to 1782 genes, and can contribute significantly to host genome content, including the transfer of DNA polymerases from virus to host [114]. Takemura hypothesized that one of these virus-to-eukaryote transfers was accompanied by the transformation of a viral factory into the nucleus [89]. This scenario is compatible with a recent more detailed analysis of the evolution of B polymerases in all cellular domains and viruses [116], in which several examples of horizontal transfer were found, likely mediated by viruses or plasmids. The NCLDV B polymerases of the Eukvir2/3 family and eukaryotic α, δ and ζ polymerases were found to be related to the B6 family of polymerases that is restricted to Aenigmarchaea, while the N-terminal part of the ε polymerase that performs leading strand synthesis is related to the B10 family that is restricted to two MAGs of the Heimdallarchaea, which have been proposed to be the immediate sisters of eukaryotic cells [117]. The restricted distributions of the B6 and B10 families, in contrast to the B1–3 families that are ancestral and widespread in archaea, suggests that they may themselves have viral or plasmid origins and defy a simple scenario of eukaryotic B polymerases being directly inherited from the widespread B1–3 polymerases of archaea.

Independently of Takemura’s viral karyogenesis hypothesis and at almost the same time, Bell proposed that the nucleus evolved from an enveloped virus ancestor similar to poxviruses or asfarvirus infecting a methanogenic euryarchaeal mycoplasmic cell, entering the cell using membrane fusion proteins and establishing a persistent viral infection. Noting that poxviruses and asfarvirus guanylyltransferases, which carry out one of three steps in capping mRNAs, diverged from eukaryotic guanylyltransferases prior to LECA, as have DNA B polymerases, the large subunit of RNA polymerases, and topoisomerases II, Bell proposed that the virus was able to maintain its linear chromosome and acquire archaeal genes, and that its membrane fusion proteins expressed on the virocell membrane were able to effect a kind of primitive phagocytosis engulfing syntrophic bacteria [118]. In a recent update [110], Bell noted that mimiviruses separate transcription from translation in viral factories that exclude ribosomes and the viral-encoded homolog of the mRNA cap-binding protein eIF4E [119], similar to the eukaryotic nucleus. These viruses encode homologs of eIF4E and the capping complex that diverged from a common ancestor with their eukaryotic homologs prior to LECA, while archaea lack homologs of these proteins, suggesting these proteins originated in viruses [110]. Bell noted that the large subunit of RNA polymerase also diverged prior to LECA, and a scenario for the origin of eukaryotic RNA polymerases from repeated transfers from NCLDV RNA polymerases to proto-eukaryotes was recently proposed [88] that is similar to Takemura’s proposal for origins of B family DNA polymerases. Indeed nearly 50 genes that form the ancestral core genetic content of NCLDVs have been inferred to be present in these viruses prior to LECA [13, 14].

An alternative scenario to the origin of the nucleus from a viral factory is that the host acquired the genes necessary to construct a nuclear membrane from viruses in order to protect itself from viral infection [111]. In this context, Takemura presented a revised hypothesis in which ancestral NCLDVs replicating in a prekaryote constructed viral factories from the endoplasmic reticulum (ER). Ancestral medusaviruses replicating near the host genome may have encompassed both the host and viral genomes in the same viral factory, to which the host responded by developing a temporary nuclear compartment from the ER to protect its genome during viral infection, leading to the eventual evolution of modern histones and RAN-mediated nuclear transport [109] in a permanent nucleus. Medusavirus would have acquired histones, polymerase δ, and RAN by horizontal gene transfer with the primitive nucleus.

A consideration for scenarios of viral karyogenesis is that current NCLDVs are generally engulfed by phagocytosing eukaryotes and are not known to infect archaea, which are often assumed to be ancestral to eukaryotes [117, 120, 121], though this latter hypothesis is disputed [105, 122–125]. Indeed horizontal gene transfers from archaea to NCLDVs are extremely rare [77] and probably occur through amoebal “melting pots” [17]. Viral karyogenesis does not depend on whether the prekaryote host was an archaeon or not, but it may depend on the prekaryote being capable of phagocytosing NCLDVs and having an endomembrane system that could be employed to construct a viral factory and an interior capsid membrane [126]. Like viral factory membranes, the nuclear envelope is continuous with and presumably derived from the ER. In deriving the nuclear membrane from the ER, the viral karyogenesis hypothesis resembles autogenous scenarios of nuclear origin [105, 127], but it adds a strong provocation for nucleus formation in order to replicate an endogenous virus that may have subsumed part or all of the host genome, or, alternatively, to protect against such viruses. Dozens of viral-encoded membrane and replication proteins are transiently present in the mimivirus viral factories despite translation occurring only outside the factory [119], suggesting regulated protein access to viral factories that might be a predecessor of nuclear access through nuclear pores regulated by the nuclear transport protein RAN.

In these scenarios, viral histones were presumably acquired from their prekaryote hosts. Selection pressure for compacting viral genomes may have led to specialization into H2B–H2A-like and H4–H3-like doublets, and may have facilitated specific doublet or heterodimer pairs in the prekaryote host through coevolutionary exchanges between NCLDVs and their hosts [25], as is proposed for DNA and RNA polymerases and actin-related proteins [21, 88, 89, 116]. After an endogenized virus transformed genes for a viral factory into genes for the primitive nucleus, these histones were sequestered in the new nucleus. The nuclear histones underwent further selection to acquire modern histone tails that enable regulation of access to the DNA, and the uncoupling of doublets may have permitted separate regulation of the individual HFDs. Alternatively, modern eukaryotic histones may have evolved directly from prekaryotic histones, and were subsequently acquired and diversified by NCLDVs from the proto-eukaryotes they infected. Viral histones may have then been selected to lose the H3α2 chaperone recognition site and conservation of their tails to avoid regulation by the nuclear genome. Other scenarios combining these features or altering the sequence of events are possible.

Among these various scenarios, we favor a scenario in which the ultimate origin of HFDs lies in LUCA, and in which viral histones in NCLDVs were acquired from prekaryote host(s) and diversified into H4–H3-like and H2B–H2A-like doublets. Since some viruses encode only H2B–H2A or only H4–H3, the possibility that these doublets evolved independently before forming nucleosome-like particles that facilitated viral genome packaging cannot be dismissed, though the occurrence of divergent triplets and quadruplets suggest the eight-HFD configuration characteristic of both marseillevirus doublet tetrasomes and eukaryotic octameric nucleosomes developed at an early stage. The large size of NCLDV genomes may have favored an ancestral packaging function for viral histones, as is suggested by the presence of histones in the virions of marseilleviruses, iridoviruses, and medusaviruses, whereas histones with long tails or additional domains suggest diversification of functional roles. The formation of the nuclear envelope, whether as part of a viral factory, in response to viral infection, or autogenously for other reasons, may have reduced horizontal gene transfers between NCLDVs and their hosts, sequestering the host genome and setting the nuclear histones on a course for individual regulation, with tails bearing PTMs to silence viruses and transposons (heterochromatic marks) or PTMs to prevent inappropriate silencing of host genes [53]. Scenarios for the origins of mitosis and meiosis under the viral karyogenesis hypothesis have been proposed [128] which can be weighed against the rapidly expanding data from NCLDVs and can be revised, rejected or expanded. Whether or not it is correct, the viral karyogenesis hypothesis has already spurred new discoveries in the biology of NCLDVs [88, 89, 110, 119] and is likely to continue to do so.

### Perspective

The discoveries of viral histones in the last two decades have led to surprising revelations. Histones co-opted by bracoviruses enter host chromatin and suppress host defenses, and may be involved in speciation of their wasp symbionts. *Marseilleviridae* histones are capable of forming nucleosomes that package viral genomes and may provide new insights into chromatin evolution. In contrast to these better studied examples, a large number of viral histones are entirely un-investigated and more are being discovered apace through metagenomic projects, which suggest that we may be only scratching the surface of viral histone diversity. Much work is needed to make sense of what these histones do and how they operate. We mostly do not know the kinds of structures they make, whether they interact with host genomes or viral genomes or both or neither, whether they are deployed in isolation from eukaryotic histones or in heteromeric combinations with them or in competition with them, or how they affect viral replication and transcription. The wide distribution of highly divergent histone doublets in NCLDVs expands the universe of chromatin and raises questions about their relationship to proto-eukaryote histones. Together with the viral karyogenesis hypothesis, they draw attention to the long-neglected role of viruses in cellular evolution and are likely to lead to new insights into the evolution of eukaryotes in particular, and viral and cellular evolution in general.

## Supplementary Information

**Additional file 1: Table S1.** Eukaryotic histones. **Table S2.** Cultured viral histones. **Table S3.** Histones from MAGs.

**Additional file 2:** **Figure S1.** Alignment of H4s from bracoviruses, *Plutella xylostella* and human. Cv: *Cotesia vestalis*, Cg: *Cotesia glomerata*, Csk: *Cotesia sesamiae kitale*, Csm: *Cotesia sesamiae Mombasa, *Cc: *Cotesia congregata.* Lysines are marked in blue. **Figure S2.** Alignment of H2Bs from pandoraviruses, A*canthamoeba castellanii, *and human. Lysines are marked in blue. Aspartic and glutamic acids are marked in red. **Figure S3.** Single viral H3s and eukaryotic H3s. Lysines are marked in blue. Aspartic and glutamic acids are marked in red. The chaperone recognition site is marked in green. A potential phosphorylation hotspot is underlined. The string of acidic residues in place of the αN helix in the H3 of invertebrate iridovirus 31 (IIV31) likely make it unable to form a nucleosome. **Figure S4.** Marseillevirus doublet histones. Marseilleviridae clades: A-blue; B-magenta; C-khaki; D-green; E-yellow. Eukaryotes-grey. **Figure S5.** Insect iridovirus H4–H3-like doublets. Lysines are marked in blue. The H3 α2 chaperone recognition site is marked in green. Arginines that contact DNA in H4 and H3 are marked with ^. **Figure S6.** Medusaviruses and Clandestinovirus Histones. **Figure S7.** Indivirus and Klosneuvirus H2A–H2B histones. **Figure S8.** Marine iridovirus histones. Figure S9. Loki’s Castle Histones.
