# Supplementary material for: Viral histones: pickpocket’s prize or primordial progenitor?
Source: Epigenetics Chromatin. 2022 May 28;15:21. doi: 10.1186/s13072-022-00454-7 (PMC9145170; doi:10.1186/s13072-022-00454-7)
Supplement: Supplementary file 2 — Additional file 2: Figure S1. Alignment of H4s from bracoviruses, Plutella xylostella and human. Cv: Cotesia vestalis, Cg: Cotesia glomerata, Csk: Cotesia sesamiae kitale, Csm: Cotesia sesamiae Mombasa, Cc: Cotesia congregata. Lysines are marked in blue. Figure S2. Alignment of H2Bs from pandoraviruses, Acanthamoeba castellanii, and human. Lysines are marked in blue. Aspartic and glutamic acids are marked in red. Figure S3. Single viral H3s and eukaryotic H3s. Lysines are marked in blue. Aspartic and glutamic acids are marked in red. The chaperone recognition site is marked in green. A potential phosphorylation hotspot is underlined. The string of acidic residues in place of the αN helix in the H3 of invertebrate iridovirus 31 (IIV31) likely make it unable to form a nucleosome. Figure S4. Marseillevirus doublet histones. Marseilleviridae clades: A-blue; B-magenta; C-khaki; D-green; E-yellow. Eukaryotes-grey. Figure S5. Insect iridovirus H4–H3-like doublets. Lysines are marked in blue. The H3 α2 chaperone recognition site is marked in green. Arginines that contact DNA in H4 and H3 are marked with ^. Figure S6. Medusaviruses and Clandestinovirus Histones. Figure S7. Indivirus and Klosneuvirus H2A–H2B histones. Figure S8. Marine iridovirus histones. Figure S9. Loki’s Castle Histones. [file 13072_2022_454_MOESM2_ESM.docx]

**Supplemental Figure S1. Alignment of H4s from bracoviruses, *Plutella xylostella* and human.** Cv: *Cotesia vestalis*, Cg: *Cotesia glomerata*, Csk: *Cotesia sesamiae kitale*, Csm: *Cotesia sesamiae Mombasa,* Cc: *Cotesia congregata.* Lysines are marked in blue.

Homo_NP_001029249.1 MS---------------------------------------------GRG-------KGG

Plutella_XP_037977688.1 MT---------------------------------------------GRG-------KGG

Cv_bracovirus_AAV98010.1 MADHPKGAEEVKGSAKAGKGLGKEGKGLGKGAKGLGKEGR-------GLG-------KGT

Cg_bracovirus_YP_009665791.1 MADHPKGAEEVKGSAKAEKGLGKEGKGLGKGAKGLGKEGR-------GLG-------KGT

Csk_bracovirus_AGL91659.1 MSDCPKDAKEEKGSGKEGKGSGKERKGFGKGGK--------------GFG-------KGG

Csm_bracovirus_AFN42307.1 MSDCPKDAKEEKGS-------GKEGKGFGKGGK--------------GFG-------KGG

Cc_bracovirus_YP_184795.1 MIDRPEGAKEGKGSSKVGKGQGKEKKGLEKGGKELEKGGKVSEKGGKGLEKGEKGSVKGG

* * **

Homo_NP_001029249.1 KGLGKGGAKRHRKVLRDNIQGITKPAIRRLARRGGVKRISGLIYEETRGVLKVFLENVIR

Plutella_XP_037977688.1 KGLGKGGAKRHRKVLRDNIQGITKPAIRRLARRGGVKRISGLIYEETRGVLKVFLENVIR

Cv_bracovirus_AAV98010.1 KGLGIAGVKRYRKVLRDNIQGITKPAIRRLARRGGVKRISGLVYEEIRDVLKIFLENVIR

Cg_bracovirus_YP_009665791.1 KGLGKAGAKRYRKVLRDNIQGITKPTIRRLARRGGVKRISGLVYEEIRDVLKIFLENVIR

Csk_bracovirus_AGL91659.1 KGLGKSGTKRYRKVCRDNIQGITKPAIRRLARRGGVKRISGLVYEEIRDVLRIFLTNTIH

Csm_bracovirus_AFN42307.1 KGLGKSGTKRYRKVCRDNIQGITKPAIRRLARRGGVKRISGLVYEEIRDVLRIFLTNTIH

Cc_bracovirus_YP_184795.1 KGLGKGGAKRHRRSLRDNIQGITKPAIRRLARRGGVKRVSGLVYEEIRDVLKIFLKTVIH

**** .*.**:*. **********:************:***:*** *.**.:** ..*.

Homo_NP_001029249.1 DAVTYTEHAKRKTVTAMDVVYALKRQGRTLYGFGG

Plutella_XP_037977688.1 DAVTYTEHAKRKTVTAMDVVYALKRQGRTLYGFGG

Cv_bracovirus_AAV98010.1 DAVIYTEHAKRKTVTAMDVVYALKHKGRMIYGYGG

Cg_bracovirus_YP_009665791.1 DAVIYTEHAKRKTVTAMDVVYALKHKGRMIYGYGG

Csk_bracovirus_AGL91659.1 DAIQYTDYARRKTVTAMDIVYALNRRGRKIYGYGG

Csm_bracovirus_AFN42307.1 DAIQYTDYARRKTVTAMDIVYALNRQGRKIYGYGG

Cc_bracovirus_YP_184795.1 DAFHYTEHAKRKTVTSMDIVYALKRQGRKIYGFGS

**. **::*.*****:**:****:..** :**:*.

**Supplemental Figure S2. Alignment of H2Bs from pandoraviruses, A*canthamoeba castellanii,* and human.** Lysines are marked in blue. Aspartic and glutamic acids are marked in red.

Homo_NP_066402 ------------------------------------------------------------

Acanthamoeba_XP_004341446.1 ------------------------------------------------------------

massiliensis_OFAI01000004 ------------------------------------------------------------

neocaledonia_YP_009482227.1 ------------------------------------------------------------

macleodensis_YP_009481299.1 ------------------------------------------------------------

braziliensis_OFAK01000003 ------------------------------------------------------------

salinus_YP_008437980.1 ------------------------------------------------------------

quercus_YP_009483470.1 ------------------------------------------------------------

celtis_QBZ81372.1 MQPCVAASPCHEATAPTVSGLWLGRERTSEKKARQRQNGGKTEPAAAVSTWLCAAGAPAQ

dulcis_YP_008319525.2 ------------------------------------------------------------

japonicus_BCU02668.1 ------------------------------------------------------------

pampulha_OFAJ01000016.1 ------------------------------------------------------------

Homo_NP_066402 ------------------------------------------------------------

Acanthamoeba_XP_004341446.1 ------------------------------------------------------------

massiliensis_OFAI01000004 ------------------------------------------------------------

neocaledonia_YP_009482227.1 ---------------------------------------------------------MSA

macleodensis_YP_009481299.1 ----------------------------------------------------------MS

braziliensis_OFAK01000003 ----------------------------------------------------------MS

salinus_YP_008437980.1 ------------------------------------------------------------

quercus_YP_009483470.1 ------------------------------------------------------------

celtis_QBZ81372.1 PLFFSRAHTRPHKKRHRPWQETLFFCAKRVHREQPTASRAACPSFFSRSIIFFFLIASDN

dulcis_YP_008319525.2 ------------------------------------------------------------

japonicus_BCU02668.1 ------------------------------------------------------------

pampulha_OFAJ01000016.1 ------------------------------------------------------------

Homo_NP_066402 -------------------MPEPAKS----------------------------APAPKK

Acanthamoeba_XP_004341446.1 --MSSTPKKTG---GK---APAKKPA-DTVE-----------------------------

massiliensis_OFAI01000004 -MSASEPLPMD---TD---APVASPPAATAAPADDAGAPEGGGAPAVHDTTTESVAKHEP

neocaledonia_YP_009482227.1 PSAASDSLPMD---TD---APVAAPP-TLEDQTSGLGTGDIG-------AAADTVAKHEP

macleodensis_YP_009481299.1 ASSASDSSPMD---TD---TPVTAPP-AIEEQVGDLGDHTDG------DASADSIAKHEP

braziliensis_OFAK01000003 ASSASDSSPMD---TD---TPVTAPP-AIEEQVGDLGDHTDG------DASADSIAKHEP

salinus_YP_008437980.1 -MSA-PAPTDIEMTTDASVPADAAP-TAAEPQHQRDMETET-------EAASAVPKPEP

quercus_YP_009483470.1 -MSAPSPSPMD---TET--VPEPQVP-AEAVPAGEAAATETG-----TEAGTAAVPKPEP

celtis_QBZ81372.1 NMSAPSPSPMD---TET--VPEPQVP-AEAVPAGEAAATETG-----TEAGTAAVPKPEP

dulcis_YP_008319525.2 -MSA--PSPMD---TNTEVAPEPQAV-ASETADNEATAPSAV-------SEADAAPKAEP

japonicus_BCU02668.1 -MSA--PSPMD---THTEVAPEPQAI-ASEAVDNEAAVPLAA-------SEADAAPKAEP

pampulha_OFAJ01000016.1 -MSA--PSPMD---THTEVAPEPQAI-ASEAVDNEAAVPLAA-------SEADAAPKAEP

*

Homo_NP_066402 GSKKAV---------------TKAQKKDGK-----KRKRSRKE----SYSIYVYKVLKQV

Acanthamoeba_XP_004341446.1 GAEKKT-TGGKKTAATKKVGVKKTAGEGKK----GTKGGKGSKKNYQSYSTFIYKVLKQV

massiliensis_OFAI01000004 GTAKAKKTIKKKKRSASGLGVSKAESVDGA-----KRKAHRHKKDYASYSTFIYRVLKQV

neocaledonia_YP_009482227.1 GAAKKTKKIAKKKKGAALGGVGKTESLDGV-----KRKTHRHKKDYASYSTFIYRVLKQV

macleodensis_YP_009481299.1 GTAKKA-KGKKTLKKKKAAGTPKTESLDGV-----KRKAYRHKKDYASYSTFIYRVLKQV

braziliensis_OFAK01000003 GTAKKT-KGKKTLKKKKAVGTAKTESLDGV-----KRKAYRHKKDYASYSTFIYRVLKQV

salinus_YP_008437980.1 GATKTKKKAAKRTKKIAL--STKKTQSGGDAGAAGKRGRGQRKKNYASYSSFVYKVLKQV

quercus_YP_009483470.1 GAKKTKKKAAKRPKKIAL--STKTEGDAGA---VGKRGRGQRKKNYASYSSFIYKVLKQV

celtis_QBZ81372.1 GAKKTKKKAAKRPKKIAL--STKTEGDAGA---VGKRGRGQRKKNYASYSSFIYKVLKQV

dulcis_YP_008319525.2 GTKATKKKAGKQGKKKIVLSTAAKA-EGGAAAKVGKRGRGQRKKNYTSYSSFIYKVLKQV

japonicus_BCU02668.1 GTKATKKKAGKQGKKKIVLSTAAKAGEGGAAAKAGKRGRGQRKKNYTSYSSFIYKVLKQV

pampulha_OFAJ01000016.1 GTKATKKKAGKQGKKKIVLSTAAKAGEGGAAAKAGKRGRGQRKKNYTSYSSFIYKVLKQV

*: .. : *** ::*.*****

Homo_NP_066402 HPDTGISSKAMGIMNSFVNDIFERIAGEASRLAHYNKRSTITSREIQTAVRLLLPGELAK

Acanthamoeba_XP_004341446.1 HPDTGISNKAMAIMNSFVNDIFERIALEAGRLARYNKRNTISSREIQTAVRLLLPGELAK

massiliensis_OFAI01000004 HPDVGISNKSMSIMNSFVNDMIDRIATEAGRLARTNKRNTITAREIQTAVRLIMQGELAR

neocaledonia_YP_009482227.1 HPDVGISNKSMSIMNSFVNDMIDRIATEAGRLARTNKRNTITAREIQTAVRLIMQGELAR

macleodensis_YP_009481299.1 HPDVGISNKSMSIMNSFVNDMIDRIATEAGRLARTNKRNTITAREIQTAVRLIMQGELAR

braziliensis_OFAK01000003 HPDVGISNKSMSIMNSFVNDMIDRIATEAGRLARTNKRNTITAREIQTAVRLIMQGELAR

salinus_YP_008437980.1 HPDVGISNKSMSIMNSFVNDMIDRIGTEAGRLARTNKRNTIGTREIQTAVRLIMRGELAR

quercus_YP_009483470.1 HPDVGISNKSMSIMNSFVNDMIDRIGTEAGRLARSNKRNTIGTREIQTAVRLIMRGELAR

celtis_QBZ81372.1 HPDVGISNKSMSIMNSFVNDMIDRIGTEAGRLARSNKRNTIGTREIQTAVRLIMRGELAR

dulcis_YP_008319525.2 HPDVGISNKSMSVMNSFVNDMIDRIGTEAGRLAHSNKRNTIGTREIQTAVRLIMRGELAR

japonicus_BCU02668.1 HPDVGISNKSMSVMNSFVNDMIDRIGTEAGRLAHSNKRNTIGTREIQTAVRLIMRGELAR

pampulha_OFAJ01000016.1 HPDVGISNKSMSVMNSFVNDMIDRIGTEAGRLAHSNKRNTIGTREIQTAVRLIMRGELAR

***.***.*:*.:*******:::**. **.***. ***.** :*********:: ****.

Homo_NP_066402 HAVSEGTKAVTKYTSAK------------------

Acanthamoeba_XP_004341446.1 HAVSEGTKAVTKYQSSLGSTAASQDE---------

massiliensis_OFAI01000004 HAVSEGTKAVTKYNDAVNNSAVADDE-----TAAA

neocaledonia_YP_009482227.1 HAVSEGTKAVTKYNEAVNAGSVGDDE-----TAAA

macleodensis_YP_009481299.1 HAVSEGTKAVTKYNEAVNAGSVGDDE-----TAAA

braziliensis_OFAK01000003 HAVSEGTKAVTKYNEAVNAGSVGDDE-----TAAA

salinus_YP_008437980.1 HAVSEGTKAVTKYNDAINAAAAEAHA-VAVDTAAA

quercus_YP_009483470.1 HAVSEGTKAVTKYNEAVNAAAEANAASAAIKSTAA

celtis_QBZ81372.1 HAVSEGTKAVTKYNEAVNAAAEANAASATIKSTAA

dulcis_YP_008319525.2 HAVSEGTKAVTKYNEAVNAAAETAAA-AAIDTTAA

japonicus_BCU02668.1 HAVSEGTKAVTKYNEAVNAATEAHAA-ATIDTTAA

pampulha_OFAJ01000016.1 HAVSEGTKAVTKYNEAVNAATEAHAA-AAIDTTAA

************* .:

**Supplemental Figure S3. Single viral H3s and eukaryotic H3s.** Lysines are marked in blue. Aspartic and glutamic acids are marked in red. The chaperone recognition site is marked in green. A potential phosphorylation hotspot is underlined. The string of acidic residues in place of the αN helix in the H3 of invertebrate iridovirus 31 (IIV31) likely make it unable to form a nucleosome.

Homo_H3.1_NP_001363866.1 ------------------------------------------------------------

Homo_H3.3_NP_005315.1 ------------------------------------------------------------

Ruditapes_H3.2_AFI61665.1 ------------------------------------------------------------

Ruditapes_H3.3_AAX19363.1 ------------------------------------------------------------

Porphyridium_KAA8495290.1 ------------------------------------------------------------

Chlorella_XP_005843095.1 ------------------------------------------------------------

Xenomavirus_DAC81234.1 ---------------------------------------MEIDLKKKKDLSSFSRMVNRE

MM12_ERX552261.23 ------------------------------------------------------------

Sylvanvirus_AYV87056.1 ------------------------------------------------------------

LP03_phycodnavirus_ERX552270.64 ------------------------------------------------------------

DSLPV1_YP_009465730.1 ---------------MRARSPARAASSMVDTLWRTRLSVTEIFLVQNLEISIFVWYKNPG

MM18_SRX310217.15 MFQIVPECLEFFLDIRKLETKRRATGIFGKKYKRGRGLQSIPPVATTTSSDSSSNTNRKT

IIV31_YP_009046636.1 ------------------------------------------------------------

Homo_H3.1_NP_001363866.1 --------MARTKQTARKSTGGKAPRKQLATK----------AARKSAPATGGVK--KPH

Homo_H3.3_NP_005315.1 --------MARTKQTARKSTGGKAPRKQLATK----------AARKSAPSTGGVK--KPH

Ruditapes_H3.2_AFI61665.1 --------MARTKQTARKSTGGKAPRKQLATK----------AARKSAPATGGVK--KPH

Ruditapes_H3.3_AAX19363.1 --------MARTKQTARKSTGGKAPRKQLATK----------AARKSAPSTGGVK--KPH

Porphyridium_KAA8495290.1 --------MARTKQTARKSTGGKAPRKHLAAK----------AARKSAPSSGGVK--KPH

Chlorella_XP_005843095.1 --------MARTKQTARKSTGGKAPRKQLATK----------ARP---------Q--KPH

Xenomavirus_DAC81234.1 TQKRKAEIEKRREILARK------PRKNVPTKVLVGQVQKTIAKKTVKKSGGGKKIFKSR

MM12_ERX552261.23 --------MARSKQTAKKSIGGFAPRK--------------------------------R

Sylvanvirus_AYV87056.1 --------MARTKQTARKSCSGKTPRKMLATK----------TARKAFPQDAGIKGKRKF

LP03_phycodnavirus_ERX552270.64 --------MARTKQTARRSTGGKAPRKDLAAK----------AARRTSTPWSQVK-PKKR

DSLPV1_YP_009465730.1 CPHNTPHTMARTKQTARKSTGGKAPRKQLACK----------AARKSQALPGGVK--KPH

MM18_SRX310217.15 YQSTRQIIMARTKQTARKSTGGKVPRKLLATK----------CPR-----TAPVK--KPH

IIV31_YP_009046636.1 ----------MEQTMNKLSLDDDEWEDEIEDE----------EEIKAEEENSEEE-----

: . .

Homo_H3.1_NP_001363866.1 RYRPGTVALREIRRYQKSTEL--------LIRKLPFQRLVREIAQD---F-KTDLRFQS

Homo_H3.3_NP_005315.1 RYRPGTVALREIRRYQKSTEL--------LIRKLPFQRLVREIAQD---F-KTDLRFQS

Ruditapes_H3.2_AFI61665.1 RYRPGTVALREIRRYQKSTEL--------LIRKLPFQRLVREIAQD---F-KTDLRLQS

Ruditapes_H3.3_AAX19363.1 RYRPGTVALREIRRYQKSTEL--------LIRKLPFQRLVREIAQD---F-KTDLRFQS

Porphyridium_KAA8495290.1 RYRPGTVALREIRKYQKSTEL--------LIRKLPFQRLVREIAQD---F-KTDLRFQS

Chlorella_XP_005843095.1 RYRPGTVALREIRKYQKSTEL--------LIRKLPFQRLVREIAQD---F-KTDLRFQS

Xenomavirus_DAC81234.1 RYRPGTVALRQIRKYQKSTEL--------LIKKMPFQRLVREIASKEAMF--RNLRFQS

MM12_ERX552261.23 RYRPGTVALREIRKFQKSTEL--------LIRKLPFQRLVREVTRD---Y-NTEIRFQS

Sylvanvirus_AYV87056.1 RYRPGTVCLQEIRRFQRSTEL--------LIKKMPFQRLVREIAEE---F-KHDLRFQS

LP03_phycodnavirus_ERX552270.64 RYRPGTVAIREIRKFQKSTEL--------LIRKLPFQRLVKEIAQD---VTSTPMRFQS

DSLPV1_YP_009465730.1 RYRPGTVAIREIRKYQKSTDL--------LIRKIPFQRLVKEIASD---F-AGDLRFQS

MM18_SRX310217.15 RYRPGTVALREIRKYQKDTSL--------LIRKLPFQRLVREIAQD---F-KTDLRFQS

IIV31_YP_009046636.1 --SESDNEVEEVEEDEEEEEITDDCGEGSFIPKTPFKRLVKEIAQD---F-AVDLRFQK

. : :: : . .: :* * **:***.*:: . :*:*.

Homo_H3.1_NP_001363866.1 SAVMALQEACEATLVGLFEDTNLCAIHAKRVTIMPKDIQ--------LARRIRGERA------

Homo_H3.3_NP_005315.1 AAIGALQEASEAYLVGLFEDTNLCAIHAKRVTIMPKDIQ--------LARRIRGERA------

Ruditapes_H3.2_AFI61665.1 SAVLALQEASEAYLVGLFEDTNLCAIHAERVTIMPKDIQ--------LARRIRGERA------

Ruditapes_H3.3_AAX19363.1 AAIGALQEASEAYLVGLFEDTNLCAIHAKRVTIMPKDIQ--------LARRIRGERA------

Porphyridium_KAA8495290.1 SAVMALQEASEAYLVGLFEDTNLCAIHAKRVTIMPKDIQ--------LARRIRGERA------

Chlorella_XP_005843095.1 SAVLALQEAAEAYLVGLFEDTNLCAIHAKRVTIMPKDIQ--------LARRIRGERA------

Xenomavirus_DAC81234.1 SAILALQEASEAYLVGLFEDAYSAALHTKRVTLMPKDIL--------LAKRIRGDQN------

MM12_ERX552261.23 LAILALQEAAEDYLVSLFQDTNLCAIHAKRVTITQKDISREAYSWYIMVESVINN--------

Sylvanvirus_AYV87056.1 TAILALQEISESYLVGLFEDTNLCAIHAKRVTISVRDMH--------LALRIRGERS------

LP03_phycodnavirus_ERX552270.64 IAILAIQEASEAFLTGLFEDTNLCAIHAKRVTIMPKDMQ--------LARRIRGNR-------

DSLPV1_YP_009465730.1 TAILALQEAAEAYLTSLFEDTNLCAIHAKRITIMPKDMQ--------LARRIRGERA------

MM18_SRX310217.15 AAIGALQEAAEAYLVGLFEDTNLACIHAKRVTITPKDMQ--------LARRLRGERC------

IIV31_YP_009046636.1 EAIDALQQVSEEYLIKLFHDANKCAEHAKRQTVTPDDIK--------LVRFLRQNKSMDFELS

*: *:*: .* * ** *: .. *::* *: *: :. : :

**Supplemental Figure S4. Marseillevirus doublet histones**. Marseilleviridae clades: A-blue; B-magenta; C-khaki; D-green; E-yellow. Eukaryotes-grey.

**Hδ-Hγ**

Homo_H4xH3.3 MSGRGKGGKGLGKGG-------AKRHRKVLRDNIQGITKPAIRRLARRGGVKRISGLIYE

Acanthamoeba_H4xH3 MSHVGRGKGGKGLGKGGKGLGGAKRHRKVLRDNIQGITKPAIRRLARRGGVKRISGMIYE

Tokyovirus_YP_009254842.1 MSKAAKKSKSQQHGH----------LASYVLGGETQIPKATTQHLLRKAGSLSATDDTEV

Marseillevirus_YP_003407137.1 MSKAGKKVKAQQHGH----------LADHVSVGETQIPKASTQHLLRKAGSLSAAGDTEV

Cannes_8_AGV01787.1 MSKAGKKVKAQQHGH----------LADHVSVGETQIPKASTQHLLRKAGSLSAAGDTEV

Melbournevirus_YP_009094869.1 MSKAGKKVKAQQHGH----------LADHVSVGETQIPKASTQHLLRKAGSLSAAGDTEV

Golden_Marseillvirus_KT835053.1 MSARA--------------------LSSFLNRGETRLPKATTQHLLKKAGARTASSDTFE

Tunisvirus_YP_009507075.1 MSKSGKKTLTRTSGH----------LSSFVHDKETQLPKATTQHLLRKAGSHTSSATAFE

Insectomime_AHA45912.1 MSKSGKKTLTRTSGH----------LSSFVHDKETQLPKATTQHLLRKAGSHTSSATAFE

Noumeavirus_YP_009345223.1 MSKSGKKSAAHAPGH----------LASFVRSKETQLPKATTQHLLRKAGARTSSASTFE

Kurlavirus_AQQ73806.1 MSKSGKKSVAHAPGH----------LASFVRSKETQLPKATTQHLLRKAGARTSSASTFE

Lausannevirus_YP_004347349.1 MSKSGKKTLAPAPGY----------LASFVRSKETQLPRATTQHLLRKAGSRTSTADTFE

Port-miou_viru_ALH07048.1 MSKSGKKTLAPAPGY----------LASFVRSKETQLPRATTQHLLRKAGSRTSTADTFE

Braz_marseillevirus_YP_009238943 MSKSGKKTLARPLGH----------LASFVHSKETQLPKATTQHLLRKAGSRTSAADAFE

** . . : :...: ..* ...* :

Homo_H4xH3.3 ETRGVLKVFLENVIRDAVTYTEHAKRKTVTAMDVVYALKRQGRTLYGFGGXMARTKQTAR

Acanthamoeba_H4xH3 ETRGVLKVFLENVIRDAVTYTEHARRKTVTAMDVVYALKRQGRTLYGFGGXMARTKQTAR

Tokyovirus_YP_009254842.1 PIRGFVRMKLHKLLQKSLLAMQLAKRKTILKGDVKKAAELMHLPVFAIPT----------

Marseillevirus_YP_003407137.1 PIRGFVHMKLHKLVQKSLLAMQLAKRKTIMKSDVKKAAELMHLPVFAIPT----------

Cannes_8_AGV01787.1 PIRGFVHMKLHKLVQKSLLAMQLAKRKTIMKSDVKKAAELMHLPVFAIPT----------

Melbournevirus_YP_009094869.1 PIRGFVHMKLHKLVQKSLLAMQLAKRKTIMKSDVKKAAELMHLPVFAIPT----------

Golden_Marseillvirus_KT835053.1 PICGYVAMKIDKILKRALMTMQYANRSTLLKGDVLKAAEMLHIPVFAVPK---------K

Tunisvirus_YP_009507075.1 PITGFVHMKLHKLLGKALLAMQYAKRTTLLKGDVVKAAEMMHLPVFAIPS---------K

Insectomime_AHA45912.1 PITGFVHMKLHKLLGKALLAMQYAKRTTLLKGDVVKAAEMMHLPVFAIPS---------K

Noumeavirus_YP_009345223.1 PITGFVHMKLEKLLDKALLSMQFAKRTTLLKEDVKKAAEMMHLPVFAIPS---------K

Kurlavirus_AQQ73806.1 PITGFVHMKLEKLLDKALLSMQFAKRTTLLKEDVKKAAEMMHLPVFAIPS---------K

Lausannevirus_YP_004347349.1 PITGFVHMKLEKLLGKALLAMQFAKRTTLLKEDVKKAAEMMHLPVFAVPS---------K

Port-miou_viru_ALH07048.1 PITGFVHMKLEKLLGKALLAMQFAKRTTLLKEDVKKAAEMMHLPVFAVPS---------K

Braz_marseillevirus_YP_009238943 PITGFVHMKLEKLLGKALLSMQFAKRTTLLKEDVQKAAEMMHLPVFAIPS---------K

* : : : ::: :: : *.*.*: ** * : .::..

Homo_H4xH3.3 KSTGGKAPRKQLATKAARKSAPSTGGVKKPHRYRPGTVALREIRRYQKSTELLIRKLPFQ

Acanthamoeba_H4xH3 KSTGGKAPR-KMASKSAKK-SALAGGVKKPHRFRPGTVALREIRKYQKSTDLLIRKAPFQ

Tokyovirus_YP_009254842.1 KESGAKGSV-FLSCRQKGAGSAGSG----------SDTNSQEVRNQMKSSCLIIPKERFR

Marseillevirus_YP_003407137.1 KDSGAKGSV-FLSCRQKGAGSAGTG----------SETNSQEVRSQMRSTCLIIPKERFR

Cannes_8_AGV01787.1 KDSGAKGSV-FLSCRQKGAGSAGTG----------SETNSQEVRSQMRSTCLIIPKERFR

Melbournevirus_YP_009094869.1 KDSGAKGSV-FLSCRQKGAGSAGTG----------SETNSQEVRSQMKSTCLIIPKERFR

Golden_Marseillvirus_KT835053.1 GESGTRGSV-FLSCRQDGSGSQLKG----------KETGTQEVRKQMKQTCLIIPKARFR

Tunisvirus_YP_009507075.1 KESGAKGSV-FLSCRQAGSGSELKG----------KETNVQEIRKQQRQTCMIIPKERFR

Insectomime_AHA45912.1 KESGAKGSV-FLSCRQAGSGSELKG----------KETNVQEIRKQQRQTCMIIPKERFR

Noumeavirus_YP_009345223.1 KESGAKGSI-FLSCRQSGSGSELKG----------KETNAQEIRKQQRQTCMIIPKARFR

Kurlavirus_AQQ73806.1 KESGAKGSI-FLSCRQSGSGSELKG----------KETNAQEIRKQQRQTCMIIPKARFR

Lausannevirus_YP_004347349.1 KESGAKGSV-FLSCRQSGSGSELKG----------KETNMQEIRKQQRQTCMIIPKARFR

Port-miou_viru_ALH07048.1 KESGAKGSV-FLSCRQSGSGSELKG----------KETNMQEIRKQQRQTCMIIPKARFR

Braz_marseillevirus_YP_009238943 KETGAKGSV-FLSCRQSGSGSELKG----------KETNVQEIRKQQRQTCMIIPKERFR

.:* ... :: . : * . .*:* ..: ::* * *.

Homo_H4xH3.3 RLVREIAQDFKT---DLRFQSAAIGALQEASEAYLVGLFEDTNLCAIHAKRVTIMPKDIQ

Acanthamoeba_H4xH3 RLVRELAQDFKT---DLRFQASAVAALQEAGEAYLVGLFEDTNLCAIHAKRVTIMPKDIQ

Tokyovirus_YP_009254842.1 TMAKEISKREGH---DVHIAEAALDMLQVIVESCTVRLLEKALVITYEKDKKRVTSRDIE

Marseillevirus_YP_003407137.1 TMAKEISKKEGH---DVHIAEAALDMLQVIVESCTVRLLEKALVITYSGKRTRVTSKDIE

Cannes_8_AGV01787.1 TMAKEISKKEGH---DVHIAEAALDMLQVIVESCTVRLLEKALVITYSGKRTRVTSKDIE

Melbournevirus_YP_009094869.1 TMAKEISKKEGH---DVHIAEAALDMLQVIVESCTVRLLEKALVITYSGKRTRVTSKDIE

Golden_Marseillvirus_KT835053.1 DIVQESAVKIGAGYADVRFSEKALDILQVIVESMTVRLLEKARILTTAGKRDRVTGKDID

Tunisvirus_YP_009507075.1 TIVKEIADKLAMV-DSVRLSEKALDLLQLIVESCTVRLLEKALALTKSAKRDRVNGQDIE

Insectomime_AHA45912.1 TIVKEIADKLAMV-DSVRLSEKALDLLQLIVESCTVRLLEKALALTKSAKRDRVNGQDIE

Noumeavirus_YP_009345223.1 DIVKEIADRQSTE-KDVRLSEKALDLLQIIVESLTVRLLEKALVLTLEAKRDRVTGHDIE

Kurlavirus_AQQ73806.1 DIVKEIADRQSTE-KDVRLSEKALDLLQIIVESLTVRLLEKALVLTLEAKRDRVTGHDIE

Lausannevirus_YP_004347349.1 DIAKEIVDRELE---GVRLSEKALDLLQLIVESLTVRLLEKAVALTLEAQKDRVTGRSIE

Port-miou_viru_ALH07048.1 DIAKEIVDRELE---GVRLSEKALDLLQLIVESLTVRLLEKAVALTLEAQKDRVTGRSIE

Braz_marseillevirus_YP_009238943 TIVKEIADKLSVA-ESVRLSEKALDLLQVIVESCTVRLLEKALALTMEAGKDRVTGRHIE

:..* .:.: *: ** *: * *:*.: : . : . *:

Homo_H4xH3.3 LARRIRGERA

Acanthamoeba_H4xH3 LARRIRGERA

Tokyovirus_YP_009254842.1 TAFMLEHGPL

Marseillevirus_YP_003407137.1 TAFMLEHGPL

Cannes_8_AGV01787.1 TAFMLEHGPL

Melbournevirus_YP_009094869.1 TAFMLEHGPL

Golden_Marseillvirus_KT835053.1 GAFFIEHGPY

Tunisvirus_YP_009507075.1 TVFLIEHGPL

Insectomime_AHA45912.1 TVFLIEHGPL

Noumeavirus_YP_009345223.1 TMFKIEHGPL

Kurlavirus_AQQ73806.1 TMFKIEHGPL

Lausannevirus_YP_004347349.1 AIFKIEHGPL

Port-miou_viru_ALH07048.1 AIFKIEHGPL

Braz_marseillevirus_YP_009238943 AIFLIEHGPL

:

**Hβ-Hα**

Homo_H2BxH2A ---------MPEPAKSAPAPKKGSKKAVTKAQ-----------------KK--DGKKRKR

Acanthamoeba_H2BxH2A MSSTPKKTGGKAPAKKPADTVEGAEKKTTGGKKTAATKKVGVKKTAGEGKKGTKGGKGSK

Tokyovirus_YP_009254841 ---------------------MATQKETTRKR----------------------------

Marseillevirus_ADB04176 ---------------------MATQKETTRKR----------------------------

Cannes8virus_AGV01788.1 ---------------------MATQKETTRKR----------------------------

Melbournevirus_YP_009094870.1 ---------------------MATQKETTRKR----------------------------

Golden_marseiilvirusBA ---------------------MASETKEKSTN--------------------------PK

Tunisvirus_YP_009507074.1 ---------------------MASKAVKQKSK-----------------KSGEASDRKKP

Insectomimevirus_AHA45913.1 ---------------------MASKAVKQKSK-----------------KSGEASDRKKP

Noumeavirus_YP_009345224.1 ---------------------MAS---TTKSK-----------------PE-GTVSRKKP

Kurlavirus_AQQ73805.1 ---------------------MASTT---KSK-----------------PE-GTVSRKKP

Lausannevirus_YP_004347348.1 ---------------------MASRTKQEKSK-----------------PE-GTVSRKKP

Port-miouvirus_ALH07047.1 ---------------------MASRTKQEKSK-----------------PE-GTVSRKKP

Brazilianmarseillevirus_YP_00923 ---------------------MASKATKEKSK-----------------KSEDASARKKP

.: .

Homo_H2BxH2A SRKESYSIYVYKVLKQVHPDTGISSKAMGIMNSFVNDIFERIAGEASRLAHYNKRSTITS

Acanthamoeba_H2BxH2A KNYQSYSTFIYKVLKQVHPDTGISNKAMAIMNSFVNDIFERIALEAGRLARYNKRNTISS

Tokyovirus_YP_009254841 DKSVNFRLGLRSLLSQIHPDISVQTEALAELSNITVFVGKKISHGAATLLPEGTK-TISA

Marseillevirus_ADB04176 DKSVNFRLGLRNMLAQIHPDISVQTEALSELSNIAVFLGKKISHGAVTLLPEGTK-TIKS

Cannes8virus_AGV01788.1 DKSVNFRLGLRNMLAQIHPDISVQTEALSELSNIAVFLGKKISHGAVTLLPEGTK-TIKS

Melbournevirus_YP_009094870.1 DKSVNFRLGLRNMLAQIHPDISVQTEALSELSNIAVFLGKKISHGAVTLLPEGTK-TIKS

Golden_marseiilvirusBA KKELNFRVGISAITKDVSPSLGTTAEALEELNNISLYIGKCVAKGAAIIVDKESK-TING

Tunisvirus_YP_009507074.1 SKDINFKVGIYRVLKQVHPDQSIRVEALEELDKIALFVGKKIAKDAAILVGSESK-TING

Insectomimevirus_AHA45913.1 SKDINFKVGIYRVLKQVHPDQSIRVEALEELDKIALFVGKKIAKDAAILVGSESK-TING

Noumeavirus_YP_009345224.1 SRDVNFEVGIRRVLAQVHPDQSIKGEAMRQLDSIAVYLGKKIAHDAAVIVGTESK-TING

Kurlavirus_AQQ73805.1 SRDVNFEVGIRRVLAQVHPDQSIKGEAMRQLDSIAVYLGKKIAHDAAVIVGTESK-TING

Lausannevirus_YP_004347348.1 SKDVNFQVGIRRVLAQVHPDQSIKGEALRQLDSIAVYLGKKIAHDAAVIVGTESK-TING

Port-miouvirus_ALH07047.1 SKDVNFQVGIRRVLAQVHPDQSIKGEALRQLDSIAVYLGKKIAHDAAVIVGTESK-TING

Brazilianmarseillevirus_YP_00923 SKDINFKVGIRRVLAQVHPDQSIRAEALEELDNIAIFLGKAISKDAAILVGTESK-TING

.. .: : : :: *. . :*: :..: : : :: * : .. **..

Homo_H2BxH2A REIQTAVRLLLPGELAKHAVSEGTKAVTKYTSA---------KXMSGR--GKQGGKAR-AK

Acanthamoeba_H2BxH2A REIQTAVRLLLPGELAKHAVSEGTKAVTKYQSSLGSTAASQDEXMSSKDAGKTTKKAEGAK

Tokyovirus_YP_009254841 EAVILSAEDLFGKDLGRHAASEARKAVASYKAS---------------------KV----S

Marseillevirus_ADB04176 SAVLLAAGDLYGKDLGRHAVGEMTKAVTRYGSA---------------------KES---K

Cannes8virus_AGV01788.1 SAVLLAAGDLYGKDLGRHAVGEMTKAVTRYGSA---------------------KES---K

Melbournevirus_YP_009094870.1 SAVLLAAGDLYGKDLGRHAVGEMTKAVTRYGSA---------------------KES---K

Golden_marseiilvirusBA RAVALAARASFPDELGDKSFEKAMGAIKTSKKA---------------------GE----A

Tunisvirus_YP_009507074.1 RAIMGATRALLPGELGKHAISDITKAITHTASA---------------------NASEEHK

Insectomimevirus_AHA45913.1 RAIMGATRALLPGELGKHAISDITKAITHTASA---------------------NASEEHK

Noumeavirus_YP_009345224.1 RAVSLAARALMGGELGKHAHSGAAKAITHYQAA---------------------GD----K

Kurlavirus_AQQ73805.1 RAVSLAARALMGGELGKHAHSGAAKAITHYQDA---------------------GD----K

Lausannevirus_YP_004347348.1 RAVLLATRALFDGELAKHAHALAGKAVTHYEAA---------------------GD----K

Port-miouvirus_ALH07047.1 RAVLLATRALFDGELAKHAHALAGKAVTHYEAA---------------------GD----K

Brazilianmarseillevirus_YP_00923 RAISSAAVALLGGELGKHAHAQAGKAITASQAG---------------------DG----K

: :. :*. :: *: .

Homo_H2BxH2A AKTRSSRAGLQFPVGR------VHRLLRKG-NYSER-VGAGAPVYLAAVLEYLTAEILEL

Acanthamoeba_H2BxH2A KAPRSAPKAKPAPAGKDAAGEGHSRSFKAGLQYASR-VGAGAPVYLAAVLEYLVAEILEL

Tokyovirus_YP_009254841 EGSRSSKAKLKLSVAR------SERVLREHSACS-R-VSGGASVALAAALEYFMAEIIEL

Marseillevirus_ADB04176 EGSRSSKAKLQISVAR------SERLLREHGGCS-R-VSEGAAVALAAAIEYFMGEVLEL

Cannes8virus_AGV01788.1 EGSRSSKAKLQISVAR------SERLLREHGGCS-R-VSEGAAVALAAAIEYFMGEVLEL

Melbournevirus_YP_009094870.1 EGSRSSKAKLQISVAR------SERLLREHGGCS-R-VSEGAAVALAAAIEYFMGEVLEL

Golden_marseiilvirusBA KGARSEKAGLMISVAR------AERLIREHGGCSLSCLLSPPGIALAAVIEYVVTEVIMA

Tunisvirus_YP_009507074.1 GESRSHKAKLQMSVAR------AERVIREE-ACSYR-VSESAGIALAAALEYLIAEIVEL

Insectomimevirus_AHA45913.1 GESRSHKAKLQMSVAR------AERVIREE-ACSYR-VSESAGIALAAALEYLIAEIVEL

Noumeavirus_YP_009345224.1 GDSRSAKARLQLSVSR------AERLIREHGGCAYR-VSATAGVALAAAIEYIIAEILEL

Kurlavirus_AQQ73805.1 GDSRSAKARLQLSVSR------AERLIREHGGCAYR-VSATAGVALAAAIEYIIAEILEL

Lausannevirus_YP_004347348.1 GDSRSAKAHLQLSVSR------AERLIREHGGCGYR-VSATAGVVLAAALEYIIAEIIEL

Port-miouvirus_ALH07047.1 GDSRSAKAHLQLSVSR------AERLIREHGGCGYR-VSATAGVVLAAALEYIIAEIIEL

Brazilianmarseillevirus_YP_00923 GESRSIKARLQLSVAR------SERLIREH-ACAYR-VSATAGIALAAVLEYIIAEIIEL

.** .... * :. . : . : ***.:**. *::

Homo_H2BxH2A AGNAARDNKKTRIIPRHLQLAIRNDEELNKLLGRVTIAQGGVL--------------PNI

Acanthamoeba_H2BxH2A AGNASRDNKRVRIVPRHIQLAVRNDEELNKLLQDVTISSGGVI--------------PNI

Tokyovirus_YP_009254841 AGNAARDSKKVRISVKHIMRAIQDDAALFHVVGKGVFSGAGVSLVQVHVPRRKP-RKTTE

Marseillevirus_ADB04176 AGNAARDSKKVRISVKHITLAIQNDAALFAVVGKGVFSGAGVSLISVPIPRKKA-RKTTE

Cannes8virus_AGV01788.1 AGNAARDSKKVRISVKHITLAIQNDAALFAVVGKGVFSGAGVSLISVPIPRKKA-RKTTE

Melbournevirus_YP_009094870.1 AGNAARDSKKVRISVKHITLAIQNDAALFAVVGKGVFSGAGVSLISVPIPRKKA-RKTTE

Golden_marseiilvirusBA AALRASDSKKVRLAVKHIQEAVHEDTELMQLLGKGVFSGAGVHLHRGERARRAPTKKPAA

Tunisvirus_YP_009507074.1 AGNAARDSKKVRVSVKHIQLAVHNDSEMMALLGKGIFSGGGVKLVSMSYSR----SKPKK

Insectomimevirus_AHA45913.1 AGNAARDSKKVRVSVKHIQLAVHNDSEMMALLGKGIFSGGGVKLVSMSYSR----SKPKK

Noumeavirus_YP_009345224.1 AGNAARDSKKVRIAVKHIQAAVQADAELFGLLGKGVFSGGGVQLVATAAIPR-R-KSPAK

Kurlavirus_AQQ73805.1 AGNAARDSKKVRIAVKHIQAAVQADAELFGLLGKGVFSGGGVQLVATAAIPR-R-KSPAK

Lausannevirus_YP_004347348.1 AGNASRDSKKVRISIKHVQLAVQNDADLFRLLGKGVFSAGGVLLVGVPAPPRAR-KSPAK

Port-miouvirus_ALH07047.1 AGNASRDSKKVRISIKHVQLAVQNDADLFRLLGKGVFSAGGVLLVGVPAPPRAR-KSPAK

Brazilianmarseillevirus_YP_00923 AGNASRDSKKVRIAVKHIQLAVQNDAELHHLLGKGIFSGGGVQLVGPQYFSVKP---PKA

*. : *.*..*: .*: *:. * : :: :: .** .

Homo_H2BxH2A QAVLLPKKTE-SHHKAKGK----------------------------------

Acanthamoeba_H2BxH2A HSVLIPKSKG-KGGKAGESL-----------------------------SQEY

Tokyovirus_YP_009254841 EKA--PKKKP-SAAKKPTKK--SQKSLSERQLAKLSKKELAKYEKEQGMSPSY

Marseillevirus_ADB04176 KEASSPKKKA-APKKKKAAS-KQKKSLSDKELAKLTKKELAKYEKEQGMSPGY

Cannes8virus_AGV01788.1 KEASSPKKKA-APKKKKAAS-KQKKSLSDKELAKLTKKELAKYEKEQGMSPGY

Melbournevirus_YP_009094870.1 KEASSPKKKA-APKKKKAAS-KQKKSLSDKELAKLTKKELAKYEKEQGMSPGY

Golden_marseiilvirusBA KEGACRQRNP-ATKKPAAKKAPAKRAQKKE-----------------------

Tunisvirus_YP_009507074.1 TAAASPKKKT-SPKKKASPA--KKKSPAKKRTVKKTR---SLKKDEEGRSPFF

Insectomimevirus_AHA45913.1 TAAASPKKKT-SPKKKASPA--KKKSPAKKRTVKKTR---SLKKDEEGRSPFF

Noumeavirus_YP_009345224.1 KAASPAKKKA-SPAKKKASPASKKKAAAKKMAMEKSK------------SPFF

Kurlavirus_AQQ73805.1 KAASPAKKKA-SPAKKKASPASKKKAAAKKMAMEKSK------------SPFF

Lausannevirus_YP_004347348.1 KAA-SPAKKA-SPKKKAASP--KKGSAVQKMALRKSKAIAALQKEREGMSPSF

Port-miouvirus_ALH07047.1 KAA-SPAKKA-SPKKKAASP--KKGSAAQKMALRKSKAIAALQKEREGMSPSF

Brazilianmarseillevirus_YP_00923 KKATSPKKKPASPKKKPASP--KKKVQKRK----------SSGKDEEGRSPSF

. *

**Hζ-Hε**

Homo_H2BxH2A ---------MPEPAKSAPAPKKGSKKAVTKAQKK-------------DGKKRKRSRK---

Acanthamoeba_H2BxH2A MSSTPKKTGGKAPAKKPADTVEGAEKKTTGGKKTAATKKVGVKKTAGEGKKGTKGGKGSK

Tokyovirus_YP_009254950.1 ---------------------------------------------------MDRVGK---

Marseillevirus_YP_003406909.1 ---------------------------------------------------MDRVGK---

Cannes8virus_AGV01521.1 ---------------------------------------------------MDRVGK---

Melbournevirus_YP_009094650.1 ---------------------------------------------------MDRVGK---

Tunisvirus_YP_009506807.1 ---------------------------------------------------MERIGN---

Insectomime_AHA46167.1 ------------------------------------------------------------

Noumeavirus_YP_009345564.1 ---------------------------------------------------MERVGK---

Kurlavirus_AQQ73899.1 ---------------------------------------------------MERVGK---

Lausannevirus_YP_004347019.1 ---------------------------------------------------MERIGK---

Port-miou_virus_ALH06745.1 ---------------------------------------------------MERIGK---

Brazilianmarseillevirus_YP_00923 ---------------------------------------------------MERVGK---

Homo_H2BxH2A ---ESYSIYVYKVLKQVHPDTGISSKAMGIMNSFVNDIFERIAGEASRLAHYNKRSTITS

Acanthamoeba_H2BxH2A KNYQSYSTFIYKVLKQVHPDTGISNKAMAIMNSFVNDIFERIALEAGRLARYNKRNTISS

Tokyovirus_YP_009254950.1 -----YGLFVKRISPK---DADVTKEALETVNNMLVFLAEKLTKQATII--VGDKKTIKH

Marseillevirus_YP_003406909.1 -----YGLFIKRISPK---DADITKESLETVNNMLVFLAEKLTKQANII---IDQKTLRH

Cannes8virus_AGV01521.1 -----YGLFIKRISPK---NADVTKEALETVNNMLVFLAEKLTKQANII---IDQKTLRH

Melbournevirus_YP_009094650.1 -----YGLFIKRISPK---DADITKESLETVNNMLVFLAEKLTKQANII---IDQKTLRH

Tunisvirus_YP_009506807.1 -----YTLFVKRMTPE---GFEVTKEAVSQINNMLVFLADKTITKALIL--LGDKKTLKH

Insectomime_AHA46167.1 ------------MTPE---GFEVTKEAVSQINNMLVFLADKTITKALIL--LGDKKTLKH

Noumeavirus_YP_009345564.1 -----YGLFIKRMTPK---DTEVTKEALEQISAMLSFLAETLLRKANIL--LGEKKTVSH

Kurlavirus_AQQ73899.1 -----YGLFIKRMTPK---DTEVTKEALEQISAMLSFLAETLLRKANIL--LGEKKTVSH

Lausannevirus_YP_004347019.1 -----YGLFIKRMTPK---DTEVTKEAVEQISSMLSFLAETLLRKSTIL--LGDKKTIKH

Port-miou_virus_ALH06745.1 -----YGLFIKRMTPK---DTEVTKEAVEQISSMLSFLAETLLRKSTIL--LGDKKTIKH

Brazilianmarseillevirus_YP_00923 -----YGLYVKRMSPK---DFDVTKEAVEQINNMLVFLADKMMTKALIL--LGDKKTLKH

: : . ::.::: :. :: : : :: : ...*:

Homo_H2BxH2A REIQTAVRLLLPGELAKHAVSEGTKAVTKYTSA---------KXMSGR--GKQGGKAR-AK

Acanthamoeba_H2BxH2A REIQTAVRLLLPGELAKHAVSEGTKAVTKYQSSLGSTAASQDEXMSSKDAGKTTKKAEGAK

Tokyovirus_YP_009254950.1 DVFLWLLTDI-QGELGKHS--------QDFANSV----------LYGG-------------

Marseillevirus_YP_003406909.1 DAFLWLLTDI-QGELGKHS--------QDFANSV----------LYGE-------------

Cannes8virus_AGV01521.1 DAFLWLLTDI-QGELGKHS--------QDFANSV----------LYGG-------------

Melbournevirus_YP_009094650.1 DAFLWLLTDI-QGELGKHS--------QDFANSV----------LYGE-------------

Tunisvirus_YP_009506807.1 DILFWLLRDI-PGELGKHG--------RDYVDSV----------LYAN-------------

Insectomime_AHA46167.1 DILFWLLRDI-PGELGKHG--------RDYVDSV----------LYAN-------------

Noumeavirus_YP_009345564.1 EVIFWLLRDI-PGELGKHS--------KDYVDSV----------LYGK-------------

Kurlavirus_AQQ73899.1 EVIFWLLRDI-PGELGKHS--------KDYVDSV----------LYGK-------------

Lausannevirus_YP_004347019.1 EVIFWLLRDI-PGELGKHS--------KDYVDSV----------VYGK-------------

Port-miou_virus_ALH06745.1 EVIFWLLRDI-PGELGKHS--------KDYVDSV----------VYGK-------------

Brazilianmarseillevirus_YP_00923 DALFWLLRDV-PGELGKHG--------KDYVDSA----------LYGN-------------

: : : ***.**. .: .: : .

Homo_H2BxH2A KTRSSRAGLQFPVGR------VHRLLRKG-NYSERVGAGAPVYLAAVLEYLTAEILELA

Acanthamoeba_H2BxH2A APRSAPKAKPAPAGKDAAGEGHSRSFKAGLQYASRVGAGAPVYLAAVLEYLVAEILELA

Tokyovirus_YP_009254950.1 ------KELVFPTKR------TENLMRK--NTCLRISQNAVRALTAILEYFCGQIMEAS

Marseillevirus_YP_003406909.1 ------KELVFPTKR------TENLMRK--NTCLRISRSAVKTLTAILEYFCGQIMEAS

Cannes8virus_AGV01521.1 ------KELVFPTKR------TENLMRK--NTCLRISQSAVKTLTAILEYFCGQIMEAS

Melbournevirus_YP_009094650.1 ------KELVFPTKR------TENLMRK--NTCLRISQSAVKTLTAILEYFCGQIMEAS

Tunisvirus_YP_009506807.1 ------KELVFPTKR------TENLIRK--KSCKRVGKSSVQALTAILEYFCREILVSS

Insectomime_AHA46167.1 ------KELVFPTKR------TENLIRK--KSCKRVGKSSVQALTAILEYFCREILVSS

Noumeavirus_YP_009345564.1 ------RELIFPTKR------TENLMRT--KTCKRVGQSAVRTLTATLEYFCREILEAS

Kurlavirus_AQQ73899.1 ------RELIFPTKR------TENLMRT--KTCKRVGQSAVRTLTATLEYFCREILEAS

Lausannevirus_YP_004347019.1 ------RELIFPTKR------TENLMRT--KTCKRVGQSAVKTLTAILEYFCREVLEAA

Port-miou_virus_ALH06745.1 ------RELIFPTKR------TENLMRT--KTCKRVGQSAVKTLTAILEYFCSEVLEAA

Brazilianmarseillevirus_YP_00923 ------KELVFPTKR------TENLMRK--KTCKRVGQSSVKTLTAILEYFCQEILNSS

*. . . :. : . *:. .: *:* ***: ::: :

Homo_H2BxH2A GNAARDNKKTRIIPRHLQLAIRNDEELNKLLGRVTIAQGGVLPNIQAVLLPKKTESHHKA

Acanthamoeba_H2BxH2A GNASRDNKRVRIVPRHIQLAVRNDEELNKLLQDVTISSGGVIPNIHSVLIPKSKGKG---

Tokyovirus_YP_009254950.1 FSQAKRNKRKRIRPFEVEAAVAKDKELYSMFGK------GVIS-----------------

Marseillevirus_YP_003406909.1 FSQAKKSKRKRIRPIDIEAAISQDKELHSMFGK------GVIS-----------------

Cannes8virus_AGV01521.1 FSQAKKSKRKRIRPIDIEAAISQDKELHSMFGK------GVIS-----------------

Melbournevirus_YP_009094650.1 FSQAKKSKRKRIRPIDIEAAISQDKELHSMFGK------GVIS-----------------

Tunisvirus_YP_009506807.1 AREAKRESRKRIKVLDIQKAVKKDMELSQVFGS------GVFS-----------------

Insectomime_AHA46167.1 AREAKRESRKRIKVLDIQKAVKKDMELSQVFGS------GVFS-----------------

Noumeavirus_YP_009345564.1 SKEAKRNSRKRIRVLDIQNSVKKDGELYKVFGS------GIFS-----------------

Kurlavirus_AQQ73899.1 SKEAKRNSRKRIRVLDIQNSVKKDGELYKVFGS------GIFS-----------------

Lausannevirus_YP_004347019.1 SKEAKRNSRKRIKVLDIQNSVKKDAELFKVFGS------GIFS-----------------

Port-miou_virus_ALH06745.1 SKEAKRNSRKRIKVLDIQNSVKKDAELFKVFGS------GIFS-----------------

Brazilianmarseillevirus_YP_00923 AREAGKESRKRIKVSDVQGAVKKDRELFQVFGS------GIFS-----------------

: ... ** :: :: :* ** .:: *::.

Homo_H2BxH2A KGK---------

Acanthamoeba_H2BxH2A -GKAGESLSQEY

Tokyovirus_YP_009254950.1 -GR---------

Marseillevirus_YP_003406909.1 -GR---------

Cannes8virus_AGV01521.1 -GR---------

Melbournevirus_YP_009094650.1 -GR---------

Tunisvirus_YP_009506807.1 -GR---------

Insectomime_AHA46167.1 -GR---------

Noumeavirus_YP_009345564.1 -GR---------

Kurlavirus_AQQ73899.1 -GR---------

Lausannevirus_YP_004347019.1 -GR---------

Port-miou_virus_ALH06745.1 -GR---------

Brazilianmarseillevirus_YP_00923 -GR---------

*.

**Supplemental Figure S5. Insect iridovirus H4-H3-like doublets.** Lysines are marked in blue. The H3 α2 chaperone recognition site is marked in green. Arginines that contact DNA in H4 and H3 are marked with ^.

^

Homo_H4xH3.3 MSGRGKGGKGLGKGGAKRHRKVLRDNIQGITKPAIRRLARRGGVKRISGLIYEETRGVLK

AmI_YP_009021202.1 -----------------------------MNDSALQRIAYKAGITRISESVYGFVREAGN

IIV22_YP_008357430.1 -----------------------------MNNSALQRLAHKAGATRVSSEVYNRARNIGD

IIV30_CCV02330.1 -----------------------------MNNSALQRLAHKAGATRVSSEVYNRARNIGD

IIV9_Wiseana_YP_004732864.1 -----------------------------MNNSALQRIAHKAGATRVSSEVYNRARSIGD

IIV25_YP_009010674.1 -----------------------------MNNSALQRIAHKAGATRVSSEVYNRARSIGD

:...*:.*:* ..* .*:* :* .* .

Homo_H4xH3.3 VFLENVIRDAVTYTEHPKRKTVTAMDVVYALKRQG-RTLYGFGGXMARTKQTARKSTGGK

AmI_YP_009021202.1 MYLRSITEYAEIYAEYEGKKTISGDHAIHAIENTGFADQYKIKDSLKPCKISGKVK----

IIV22_YP_008357430.1 QYLKSIVNYAIIYCEHENKKIITEEHALHAIEHVGFSGMYRAVGDIKKCKTSNKKK----

IIV30_CCV02330.1 QYLKSIVNYAIIYCEHENKKIITEEHALHAIEHVGFSGMYRAVGDIKKCKTSNKKK----

IIV9_Wiseana_YP_004732864.1 QYLDSIVRYAIIYCDHEKKKVVTEDHALHAIEHVGFSGMYRVSGDTPKCKTSNKKK----

IIV25_YP_009010674.1 QYLDSIVRYAIIYCDHEKKKVVTEDHALHAIEHVGFSGMYRVSGDTPKCKTSNKKK----

:* .: * * :: .* :: .::*::. * * . * : . .

^ ^

Homo_H4xH3.3 APRKQLATKAARKSAPSTGGVKKPHRYRPGTVALREIRRYQKSTE-LLIRKLPFQRLVRE

AmI_YP_009021202.1 --------------------------------VLAKIREYQKQHDCNTLAKAPIERDIKS

IIV22_YP_008357430.1 --------------------------------LITRIKEYQNQSDCVTLSKAPIEQQIKS

IIV30_CCV02330.1 --------------------------------LITRIKEYQNQSDCVTLSKAPIEQQIKS

IIV9_Wiseana_YP_004732864.1 --------------------------------LVTRIKEYQNQSDCVTLSKAPIEHQIKS

IIV25_YP_009010674.1 --------------------------------LITRIKEYQNQSDCVTLSKAPIEHQIKS

: *. **:. : : * *::. :..

^

Homo_H4xH3.3 IAQDFKTDLRFQSAAIGALQEASEAYLVGLFEDTNLCAIHAKRVTIMPKDIQLARRIRGE

AmI_YP_009021202.1 F-THFK----ISKEGVQNIHSALEYFIYKLFFSALLITLNAKRKTVSVDDVKLTLRMIND

IIV22_YP_008357430.1 LGSGFK----WSKEALINIQFALEFMLYQLLYSALKVTINAKRITMLDSDVDLTIDLITT

IIV30_CCV02330.1 LGSGFK----WSKEALINIQFALEFMLYQLLYSALKVTINAKRITMLDSDVDLTIDLITT

IIV9_Wiseana_YP_004732864.1 FGSGYK----WSKESLINIQFALEFMLYQLLFSALKVTINAKRVTMSESDLDLTIDLITT

IIV25_YP_009010674.1 LGSGYK----WSKESLINIQFALEFMLYQLLSSALKVTINAKRVTMSESDLDLTIDLITT

: :* .. .: :: * * : *: .: :::*** *: .*:.*: :

Homo_H4xH3.3 RA------

AmI_YP_009021202.1 NCKSINFR

IIV22_YP_008357430.1 NCKNIRL-

IIV30_CCV02330.1 NCKNIRL-

IIV9_Wiseana_YP_004732864.1 NCKNIRI-

IIV25_YP_009010674.1 NCKNIRI-

..

**Supplemental Figure S6. Medusaviruses and Clandestinovirus Histones.**

**H4.** Lysines are marked in blue. The arginine that contacts DNA in H4 is marked with ^

Homo_H4_NP_001029249.1 --MSGRGKGGK---------GLGKGGAKRHRKVLRDNIQGITKPAIR-----RLARRGGVK-

Acanthamoeba_H4_XP_004336819.1 MSHVGRGKGGKGLGKG----GKGLGGAKRHRKVLRDNIQGITKPAIR-----RLARRGGVK-

ClandestinovirusH4_QYA18737.1 MPANNKSAAAKTTTKSKKTTQKGSGNVRNLRRRSENTVDHLKSVLPRARVSSKLAPRAGNR-

MedusavirusH4_BBI30394.1 --------------------MPKKIAARRSSKHIKNLGEEIGNSAVR-----KTVLRTGVVF

MedusavirussthenoH4moiety_QPB444 ------------------------AGASRRSGVTKSLAEEIGKSAVK-----KTALRVNAL-

. . . : : . . . . * .

^

Homo_H4_NP_001029249.1 RISGLIYEETRGVLKVFLENVIRDAVTYTE----HAKRKTVTAMDVVYALKRQGRTLYGFGG

Acanthamoeba_H4_XP_004336819.1 RISGMIYEETRGVLKVFLENVIRDAVTYTE----HARRKTVTAMDVVYALKRQGRTLYGFGG

ClandestinovirusH4_QYA18737.1 RVKSSVYP----LLKAFVDNIVKDHCERARIVSTHNGRMTIQGKDIVLAHRTTGGRFYYA--

MedusavirusH4_BBI30394.1 RLDKTVRPKFHKVMLSKLYEAVNIAKLAAK----HSGRSTIQPKDVRLGLKLASIKLLA---

MedusavirussthenoH4moiety_QPB444 RLNESVYSAFHQVMLGKLSKAVGIAMAAAV----HAGRQTIQTKDARLGLKLAGIKLLA---

*:. : :: : : : : * * *: * . . . :

**H3.** Lysines are marked in blue. The H3 α2 chaperone recognition site is marked in green. Arginines that contact DNA in H3 are marked with ^

^

Homo_H3.3_NP_005315.1 MARTKQTAR--KSTGGKAPRKQLATKAARKSAPSTGGVKKPHRYRPGTVALREIRRYQK-

Acanthamoeba_H3_XP_004342027.1 MARTKQTAR--KSTGGKAPRK-MASKSAKKSALA-GGVKKPHRFRPGTVALREIRKYQK-

ClandestinovirusH3_QYA18687.1 MARTKQVAR-KETTPKKVEKAAAEIKKP--------EIKKPHRYRPGTVALRRIHKEQK-

MedusavirusH3_BBI30395.1 MPKERAIKK-SKSAVKKVAEKKAAIKSKAKKAAT--GVKKPHRFRPGTTAKRLSKKEQKL

MedusavirussthenoH3moiety_QPB444 MPKERAIKKSKKPAAASAAKKKSASKSPAKKAGL--GVKKPHRFRPGTTAKRLAKKEQKL

*.. . . :.: .. * :*****:****.* * .. **

^ ^

Homo_H3.3_NP_005315.1 -STELLIRKLPFQRLVREIAQDFKTD-------------LRFQSAAIGALQEASEAYLVG

Acanthamoeba_H3_XP_004342027.1 -STDLLIRKAPFQRLVRELAQDFKTD-------------LRFQASAVAALQEAGEAYLVG

ClandestinovirusH3_QYA18687.1 -KYDSIVRTSPLRRLMRERLNTDLAE---------GQKGFHLSPSAVAAMSEIVDSTLLK

MedusavirusH3_BBI30395.1 SSTKTTVRRAPFGRIVRTIASLSSAD------------SMRFSANAVDLLQQGIELYMLD

MedusavirussthenoH3moiety_QPB444 SSTKTTVRRAPFGRIVRAIAARSASQISGASGKTIDLLAMRFSADAVDMLQQGVEQYMLG

. . :* *: *::* :: :.:.. *: :.: : ::

Homo_H3.3_NP_005315.1 LFEDTNLCAIH-AKRVTIMPKDIQL-------------------------ARRIRGERA--

Acanthamoeba_H3_XP_004342027.1 LFEDTNLCAIH-AKRVTIMPKDIQL-------------------------ARRIRGERA--

ClandestinovirusH3_QYA18687.1 LLHQSQKVASHNGDRITIFPKDVKL-------------------------AADLTGVKKYL

MedusavirusH3_BBI30395.1 LMKNAALAAKQ-AKRMTLMGKDIDLIQTANHEMIDEAHAAKLANTSSAGFARRRVTKKE--

MedusavirussthenoH3moiety_QPB444 LMKSAALAATQ-AKRTTLMGKDITLIRTANHEIIDEADDAKLAAASSGNALRRVASKKA--

*: .: * : ..* *:: **: * .

**H2B.** Lysines are marked in blue. Acidic residues are marked in red.

Homo_H2B_NP_066402 -------------MPEPAKSAPAPKKGS--------------------------------

AcanthamoebaH2B_XP_004341446.1 ----MSSTPKKTGGKAPAKKPADTVEGAEKKTTGGKKTAAT-------------------

ClandestinovirusH2Bmoiety_QYA187 --------------MSAEQQTPNTTEQTEQVKVDETAPQQT-------------------

MedusavirusH2B_BBI30201.1 MSQIDEHVTEMTEFEAEHESTYSEHSDEEEQELGARVPSRK-----------QKGKAAAK

MedusavirussthenoH2B_QPB44246.1 MSQIDEHVTEMTEYEAEHESQYSDHSDEEEQELGARVSPAKSASEKKKSASLRKKKSGSP

:. .

Homo_H2B_NP_066402 -KKAVTKAQKKDGKKRKRS------RKESYSIYVYKVLKQV----------HPDTGISSK

AcanthamoebaH2B_XP_004341446.1 -KKVGVKKTAGEGKKGTKGGKGSKKNYQSYSTFIYKVLKQV----------HPDTGISNK

ClandestinovirusH2Bmoiety_QYA187 EVKSDVKTEEKKTKKGRKS------NSQQMTRFVRKVLKVL----------NGDAQVSKE

MedusavirusH2B_BBI30201.1 KVKKAVAKKSGEERRRKK-------NYDSFATFIAKLVGPN------GK--GRKPGFSAK

MedusavirussthenoH2B_QPB44246.1 KKKAGVKKPSGEERKRKK-------NYDSFATFIAKLVSANTVEDKDGKKHTRKPGFSAK

* . . .. . . :. : :: *:: .. .* :

Homo_H2B_NP_066402 AMGIMNSFVNDIFERIAGEASRLAH-YNKRSTITSREIQTAVRL---LLPGE--LAKHAV

AcanthamoebaH2B_XP_004341446.1 AMAIMNSFVNDIFERIALEAGRLAR-YNKRNTISSREIQTAVRL---LLPGE--LAKHAV

ClandestinovirusH2Bmoiety_QYA187 SCQMVNAIIVGKVEEIGNICALLLRDSTHKKTLKLNTLEAALSI---HFKDSKEILKRAL

MedusavirusH2B_BBI30201.1 GMEVLESIVKSLATEMTIVANELAK-HQGRQTLGAGDFRTALAVRGSLIAREPATVKALT

MedusavirussthenoH2B_QPB44246.1 GMEVLESVVKSLATEMTVVANELAK-HQGRQTLDSRDFRTALAVRGGILAREPGTVKTLI

. ::::.: . : . * . ..*: : :*: : : . *

Homo_H2B_NP_066402 SEGTKAVTKYTSAKX--------------------

AcanthamoebaH2B_XP_004341446.1 SEGTKAVTKYQSSLGSTAASQDE------------

ClandestinovirusH2Bmoiety_QYA187 AAGALAVQKHSAYVPVKEEGKKTATPKNASKKA--

MedusavirusH2B_BBI30201.1 EMGEKAVLKYQSSLG-RPA-KTAPKKKKATKKASA

MedusavirussthenoH2B_QPB44246.1 AEGEKAVLKYNASLG-RTAKKAAPKKKKAAAH---

**H2A.** Lysines are marked in blue. Acidic residues are marked in red.

Homo_H2A_NP_066390.1 ----------------------------------MSGR--GKQGGKAR-AKAKTRSS---

AcanthamoebaH2A.X_XP_004335958.1 ----------------------------------MSSKDAGKTTKKAEGAKKAPRSA---

ClandestinovirusH2Amoiety_QYA187 ------------------------------------------------------------

MedusavirusH2A_BBI30458.1 MEIDSHVQPAEVLAAASESMQLEEQTQLPASAAGEALEELQTEGKKTSPAKKRTSSG-KN

MedusavirussthenoH2A_QPB44482.1 MEIESHVQPAEVLAAASESMQLEEQTQLPAFVAGEALEELQTEGKKTSPAKKRSAASGKN

Homo_H2A_NP_066390.1 ------RAGLQFPVGR------VHRLLRKG-NYSERVGAGAP---VYLAAVLEYLTAEIL

AcanthamoebaH2A.X_XP_004335958.1 ------PKAKPAPAGKDAAGEGHSRSFKAGLQYASRVGAGAP---VYLAAVLEYLVAEIL

ClandestinovirusH2Amoiety_QYA187 -----KKALLQLPVKR------IGKLLRTF-TNVPQMSQGVS---VFLTGAMEYYATELF

MedusavirussthenoH2A_QPB44482.1 VKRANERAGLKLPPGR------IQKIIKS--NQTVDVGRSSPTASVFLTAVIEDIVKHII

MedusavirusH2A_BBI30458.1 VKRANERAGLKLPPGR------IQKIIKA--NQTTDVGRSSPTASVFLTAVIEDIVKEII

* . . :. :. . . *:*:..:* . ::

Homo_H2A_NP_066390.1 ELAGNAARDNKKTRIIPRHLQLAIRNDEE-LNKLLGRVTIAQG---GVLPNIQAVL----

AcanthamoebaH2A.X_XP_004335958.1 ELAGNASRDNKRVRIVPRHIQLAVRNDEE-LNKLLQDVTISSG---GVIPNIHSVL----

ClandestinovirusH2Amoiety_QYA187 INALEKCNNDGKKIISPRHFPLGLQADAE-MQETLQDAILGCGTGHGVIPGIVPAV---E

MedusavirusH2A_BBI30458.1 KGADKKSEERGRIRISPQDILKYLTENGEAYMHILGDAFVSHG-GVGQVAEMAAAAANTG

MedusavirussthenoH2A_QPB44482.1 VGADKKSAERGRIRISPQDINKYLTEDGEAYMRILGDAFVAHG-GVGQVADAAVAA---G

* : . : . * *. : : : * * . :. * * :. .

Homo_H2A_NP_066390.1 ------------------LPKKTESH---------HKAKGK----------------------

AcanthamoebaH2A.X_XP_004335958.1 ------------------IPKS-------------KGKGGKAGESLSQEY-------------

ClandestinovirusH2Amoiety_QYA187 KNAYKKALLEKEQGMIDEIPADLVAQEKKRVQKLKESKKRKAAEATGEEKAAKKQKVAE-ASA

MedusavirusH2A_BBI30458.1 IKKRKRAASTAEGAPKKKIAKKAAAKKATGAKKVVKKKSGSTKSKTTGKSVTKKASSRKVASA

MedusavirussthenoH2A_QPB44482.1 TKKRKRAASGAEGAAKK-AKKATGVKPKKTIKKVVKKKSGSAKSKTAGKSVTKKTSTRK-ASA

.

**Supplemental Figure S7. Indivirus and Klosneuvirus H2A-H2B histones**

Homo_H2AxH2B MSGRGKQGGKARAKAKTRSSRAGLQFPVGRVHRLLRKG----NYSERVGAGAPVYLAAVL

Klosneuvirus_H2A-H2B ----------MVWRRNPLGSKAGLQFSVEKFDEILHK-----HNIYNLSKEDRIFVTAIA

Indivirus_H2A-H2B -----------------MEVQCGLQMSVNAIKKILINELELRGITYNISNDVAIRITAII

..***:.* . :* : .:. : ::*:

Homo_H2AxH2B EYLTAEILELAGNAARDNKKTRIIPRHLQLAIRNDEELNKLLGRVTIAQGGVLPNIQAVL

Klosneuvirus_H2A-H2B EDLVSRLYEASGN---NANGKRIYI----------EDLDQVI------------------

Indivirus_H2A-H2B EYLVAEIMELGGNVTLNKNRKRLSIDHVILAIQTDSELNKLY------------------

* *.: : * .** : : .*: .:*:::

Homo_H2AxH2B LPKKTESHHKAKGKXMPEPAKSAPAPKKGSKKAVTKAQKKDGKKRKRSRKESYSIYVYKV

Klosneuvirus_H2A-H2B ----------------------------KTYKSDIFAEVRDKLNGTADTFISMDSWSYRK

Indivirus_H2A-H2B ----------------------------NSTNNIISNKPKDETPR-------LSTWTCKI

: : : .* . : .

Homo_H2AxH2B LKQVHPDTGISSKAMGIMNSFVNDIFERIAGEASRLAHYNKRSTITSREIQTAVRLLLPG

Klosneuvirus_H2A-H2B LKQVHPELSRTKLIAFYMDYLIDSYCEKL-------AKLDLK------NLTNSVNNIFGD

Indivirus_H2A-H2B LTQIHPDTNISRDAKRFIDKLVYDTVKKF----SDMFPLNEKDI----NINDMIDLVIPN

*.*:**: . : :: :: . :.: : . :: : :: .

Homo_H2AxH2B E--LAKHAVSEGTKAVTKYTSAK

Klosneuvirus_H2A-H2B DNVLCQNILNAGKSAMDLYKKYN

Indivirus_H2A-H2B E--LSKHAKNEMNKAIAKFNSK-

: *.:: . ..*: :..

**Supplemental Figure S8. Marine iridovirus histones.**

**H2B-H2A**

Homo_H2BxH2A ------------------------------------------------------------

IR01_SRX802077.164_contig_168297 ------------------------------------------------------------

IR01_SRX802076.27_contig_36517_2 ------------------------------------------------------------

IR01_SRX803008.97_contig_12425_4 ------------------------------------------------------------

IR01_SRX803008.97_contig_1723_20 ------------------------------------------------------------

IR01_SRX802202.32_contig_1595_29 MTDGISNNAIKQLALKGSVGSLSSPVYTTIRAEIVGTIREIAEKVIGLCKMRGSKTINHE

Homo_H2BxH2A ------------------------------------------------------------

IR01_SRX802077.164_contig_168297 ------------------------------------------------------------

IR01_SRX802076.27_contig_36517_2 ------------------------------------------------------------

IR01_SRX803008.97_contig_12425_4 ------------------------------------------------------------

IR01_SRX803008.97_contig_1723_20 ------------------------------------------------------------

IR01_SRX802202.32_contig_1595_29 DVQMAISVIPYLQYMKLNKRTGTQRNVKSCRSIATNTYGMKDRAKIAKKRIKKSSESSNC

Homo_H2BxH2A ------------------------------------------MPEPAKSAPAPKKGSKKA

IR01_SRX802077.164_contig_168297 ------------------------------------------------------------

IR01_SRX802076.27_contig_36517_2 ------------------------------------------------------------

IR01_SRX803008.97_contig_12425_4 ------------------------------------------------------------

IR01_SRX803008.97_contig_1723_20 ------------------------------------------------------------

IR01_SRX802202.32_contig_1595_29 FNLPKAAIAKLFSSSTANRKKTKTAMILIQESVETHIITIFDYASRMSEKKIMKDRTVRS

Homo_H2BxH2A VTKAQKKDGKKRKRSRKESYSIYVYKVLKQVHPD-TGISSKAMGI--MNSFVNDIFERIA

IR01_SRX802077.164_contig_168297 --------------MKEINFKNYILTVLNQVLPD-TQISISASTKEVVNSLLNNIGNRIS

IR01_SRX802076.27_contig_36517_2 -----------------MSLRTYIRKQLKLIASE-SRISSVAIGE--IEALLINALEKVV

IR01_SRX803008.97_contig_12425_4 -----------------MSLRTYIRKQLKLIASE-SRISSVAIGE--IEALLINALEKVV

IR01_SRX803008.97_contig_1723_20 ----------MSNTIMHIDLKTGIRSVSKQVNLDMNRVSSEFLSE--INDICEKLLTVLA

IR01_SRX802202.32_contig_1595_29 AIHLVKNQCGSTDDHHRERLKSYFKRVYSGANVE-CKMTAEASYQ--LDIILNLVANKYA

. . : :: :: :

Homo_H2BxH2A GEASRLAHYNKRSTITSREIQTAVRLLLPGELAKHAVSEGTKAVTKYTSAKXMSGRGKQG

IR01_SRX802077.164_contig_168297 NEAVFLTTNCGEKTVTKRAMTASIRLLLTPFIGKHAIKQGLKACKKYE---------KSD

IR01_SRX802076.27_contig_36517_2 MNAIVIKMNCRKRTVEARDIEASVKVSLPSEISKYGRSRALKAYTRYN-----------A

IR01_SRX803008.97_contig_12425_4 MNAIVIKMNCRKRTVEARDIEASVKVSLPSEISKYGRSRALKAYTRYN-----------A

IR01_SRX803008.97_contig_1723_20 RTSVTIKKNCKRVEISASDIQAATNVILPATLSEFAIREGNKATFKFV------------

IR01_SRX802202.32_contig_1595_29 STAVKLCRLNKKSTVQSFHVMQASNMFMTGKLKK----TCNQEIARYR------------

: : : : : .: :. : : : .:

Homo_H2BxH2A GKARAKAKTRSSRAGLQFPVGRVHRLLRK-----GNYSERVGAGAPVYLAAVLEYLTAEI

IR01_SRX802077.164_contig_168297 TGSKSNKITAAKRSGLVFPPSRGRNLIEH----NAPKGINISQLAGVYLAGVLEFLTADI

IR01_SRX802076.27_contig_36517_2 TTVHPKGTSRSTKAGLDIPVSIVENMIRSMVADNEEKGLRVSDESGVYAGGVLQYLTIEL

IR01_SRX803008.97_contig_12425_4 TTVHPKGTSRSTKAGLDIPVSIVENMIRSMVADNEEKGLRVSDESGVYAGGVLQYLTIEL

IR01_SRX803008.97_contig_1723_20 -SNDDMKGSRSARAGLVISVPKVDSMFRYIAGNKIPN--RFSQGSIIFMTAVIEYVIAEL

IR01_SRX802202.32_contig_1595_29 NMSHDLSKKR-----LIMPSTRCGRFISK------PYSIRTSVPAKIYLTVILEYILCEM

. * :. :: . . : :: ::::: ::

Homo_H2BxH2A LELAGNAARDNKKTRIIPRHLQLAIRNDEELNKLLGRVTIAQGGVLPNIQAVLLPKKTESHHKAKGK

IR01_SRX802077.164_contig_168297 LELAGNVARDNRRSTITPRHLHLAIRLDEEFNMLFGKTGIAGSGVIPNIHAVLLQKK-----KPKKK

IR01_SRX802076.27_contig_36517_2 LEVSLHEADKENKKTITDTIVVNAVKKDIDLGELFHCRYFE-----PSRMDFVVCN-----------

IR01_SRX803008.97_contig_12425_4 LEVSLHEADKENKKTITDTIVVNAVKKDIDLGELFHCRYFE-----PSRMDFVVCN-----------

IR01_SRX803008.97_contig_1723_20 CEVSGREARSINATTIFKRHLDIAVEKDEELMNFLKIMTAM-----S--------------------

IR01_SRX802202.32_contig_1595_29 VDAASTIANGEKKVTVEPKHLQRGLYGDEELRSFMKHIGYK----IP--LSGVYRE-----------

: : * . : : .: * :: :: .

**H4-H3**

Homo_H4xH3.3 MSGRGKGGKGLGKGGAKRHRKVLRDNIQGITKPAIRRLARRGGVKRISGLIYEETRGVLK

IR01_SRX802077.164_contig_168297 ---------------MKKAKKILKDSIQGITKPAMRRLAHVGGVKTMSGIIYEDIRSILK

*. .*:*.*.********:****. **** :**:***: *.:**

Homo_H4xH3.3 VFLENVIRDAVTYTEHAKRKTVTAMDVVYALKRQG-RTLYGFGGXMARTKQTARKSTGGK

IR01_SRX802077.164_contig_168297 IFLEKQVYNSLVYTDYCKRKTVSVKDVENALSRHGNHTVIG--------KAAAKAC---K

:***: : :::.**::.*****:. ** **.*:* .*: * * :*. . *

Homo_H4xH3.3 APRKQLATKAARKSAPSTGGVKKPHRYRPGTVALREIRRYQKSTE-LLIRKLPFQRLVRE

IR01_SRX802077.164_contig_168297 SPKRK---------------TTKQNKSRPGTVSLLNIRFYQKQSNCLHIPAAAFKRLVKE

:*..: ..* :. *****:* :** ***.:: * * .*:***.*

Homo_H4xH3.3 IAQDFKT--DLRFQSAAIGALQEASEAYLVGLFEDTNLCAIHAKRVTIMPKDIQLARRIR

IR01_SRX802077.164_contig_168297 IAQDYKTIPDARFSKDALTLLQYATERYLSGLLEDANLQAIHAGRTTLHNKDIGMAKRIR

****:** * **.. *: ** *:* ** **:**:** **** *.*: *** :*.***

Homo_H4xH3.3 GERA

IR01_SRX802077.164_contig_168297 GE--

**

**H2B-H2A-H3**

human_H2BxH2AxH3 MPEPAKSAPAPKKGSKKAVTKAQKKDGKKRKRSRKESYSIYVYKVLKQVHPDTGISSKAM

LCMAC102_QBK86552.1 ---------------MATTQKKKKTKSQHPLPKGQIDFRSYISKLLHQVHPDMGMNQISK

IR01_SRX802077.164_contig_92501 -------------------MTSRKKQSRH--------FEIYVLKLLKQQYPDNSLNTNTK

IR01_SRX802202.41_contig_2978_4 -----------------------MLKGKN--------YDQYLSKLLKRVSPTNGITSNSK

IR01_SRX802963.105_contig_12868_ -----------------------MLKGKN--------YDQYLSKLLKRVSPTNGITSNSK

IR01_SRX802143.125_contig_8432_8 --------------METPNKVIRKKSSRV--------FETYISKILKTISSSNGITANSK

... : *: *:*: . .:. :

human_H2BxH2AxH3 GIMNSFVNDIFERIAGEASRLAHYNKRSTITSREIQTAVRLLLPGE-------LAKHAVS

LCMAC102_QBK86552.1 DELNAAINYIGEALTEKAVQLTDQSNRVTVSASDIQSAMFLVLPEE-------LGRHAKG

IR01_SRX802077.164_contig_92501 QQLNSVLCHLATRISTDAKSSCLHAGRRTITHIDVIVSSKMLMSDEDRLSLDIAVNEMYN

IR01_SRX802202.41_contig_2978_4 QQLCSVLCYITRVISYTVFELLSVTNKRTISDKEIIKSIIILFPKE-------LAKTMIS

IR01_SRX802963.105_contig_12868_ QQLCSVLCYITRVISYTVFELLSVTNKRTISDKEIIKSIIILFPKE-------LAKTMIS

IR01_SRX802143.125_contig_8432_8 QQTNSVLSAVSKLICEKVFILTEISKKKTISDKEIRNSIKILFPAK-------LANSIIE

: : : : . . *:: :: : :::. : .

human_H2BxH2AxH3 EGTKAVTKYTSAKXMSGRGKQGGKARAKAKTRSSRAGLQFPVGRVHRLLRKGNYSE-RVG

LCMAC102_QBK86552.1 EGGKSVTKFLKSK---GEGER-T-------TAASRAGLLFPPSRAKRFF--ANYKK-RVS

IR01_SRX802077.164_contig_92501 TIDKSKETTDAVKDDLNEDVKGG-------RREVKLGLVFPPATAEKFLREKGSSGLMVA

IR01_SRX802202.41_contig_2978_4 MCEQSIENFTKVN------ELGV-------TKQDRAGIIFPPSISEKYIRKFGMSKIMVS

IR01_SRX802963.105_contig_12868_ MCEQSIENFTKVN------ELGV-------TKQDRAGIIFPPSISEKYIRKFGMSKIMVS

IR01_SRX802143.125_contig_8432_8 NGDIAIDNFNN-----KELSKGV-------SRQDKACIIFPPSQSEKFLRNFGYSKTMVT

: . . : ** . . : . . *

human_H2BxH2AxH3 AGAPVYLAAVLEYLTAEILELAGNAARDNKKTRIIPRHLQLAIRNDEELNKLLGRVTIA-

LCMAC102_QBK86552.1 ETAPVYLAAVLDYLSAEILVVAGTAVRDFKKNRINVKYLYLAVTHDVELCELFGKLDIQL

IR01_SRX802077.164_contig_92501 KKASVILCVVLETIMRDILTQSVAQM-NTKKKRLTIQDVNRAVTTTPSLKNLFRKCTIQF

IR01_SRX802202.41_contig_2978_4 NTSSITLVTAIECLAEEILEISSLSAKQNKRVRITIRDLEIGVRTDKDICKFFVDNKISF

IR01_SRX802963.105_contig_12868_ NTSSITLVTAIECLAEEILEISSLSAKQNKRVRITIRDLEIGVRTDKDICKFFVDNKISF

IR01_SRX802143.125_contig_8432_8 SNAPVFLAGSLEYFATIILKNAVEQARNNKRVRVTIRDLEIAVRNDEDMDMFFTMNKICF

:.: * :: : ** : : *. *: . : .: .: :: *

human_H2BxH2AxH3 -QGGVLPNIQAVLLPKKTESHHKAKGKXMARTKQTARKSTGGKAPRKQLATKAARKSAPS

LCMAC102_QBK86552.1 SGEGVIPYIHPSLLEKKKKS-------------KTTRKAAEGE-----------------

IR01_SRX802077.164_contig_92501 IGGGPTDFIHPMLSVKTGKS-------------KHDVYADDD------------------

IR01_SRX802202.41_contig_2978_4 LGGGVIPCIHPKLLNNKNLP-------------QKF------------------------

IR01_SRX802963.105_contig_12868_ LGGGVIPCIHPKLLNNKNLP-------------QKF------------------------

IR01_SRX802143.125_contig_8432_8 LGGGVKPFIYHSLLKKKNRK-------------KKVVKKSDDL---------------TE

* * * :. :

human_H2BxH2AxH3 TGGVKKPHRYRPGTVALREIRRYQK-STELLIRKLPFQRLVREIAQDFKT----DLRFQS

LCMAC102_QBK86552.1 ----KLPHKYRAGTVALREIKKHQKDSGCVYFAKIAFERLVREIAQDFDI----NMRFSE

IR01_SRX802077.164_contig_92501 ----TKTRRYNPGTLVLKEIKKLQKVYNKKIIAKVPFDKAVREVFTEKNC----DSKISK

IR01_SRX802202.41_contig_2978_4 ----KNQTRYRPGVISLRNIKRVQKTSNCLILSKLPFERYTRFKLKEYQPYSDKTVKVSK

IR01_SRX802963.105_contig_12868_ ----KNQTRYRPGVISLRNIKRVQKTSNCLILSKLPFERYTRFKLKEYQPYSDKTVKVSK

IR01_SRX802143.125_contig_8432_8 TETNKKKHRFRPGTVSIREIKKFQKLSNSLTFAKFPFEKILRETINKLNT-ENTTVKISK

. .:..*.: :.:*.. ** : *..*:. * . . ....

human_H2BxH2AxH3 AAIGALQEASEAYLVGLFEDTNLCAIHAKRVTIMPKDIQLARRIRGERA-----------

LCMAC102_QBK86552.1 DAFIAFQTYMEQFLVRLLQEANLEAIHAGRVRVYPRDIQLARRIRGDSGC----------

IR01_SRX802077.164_contig_92501 QVFTVLQYYLESYLVDILHQSNLAALHAGRLKVIPEDIDFVHSIRNNLLP----------

IR01_SRX802202.41_contig_2978_4 DVFIILQHFVEQRIISLLKKAGMLVIHAGRLKLMAADINLVKIIDSGHNN----------

IR01_SRX802963.105_contig_12868_ DVFIILQHFVEQRIISLLKKAGMLVIHAGRLKLMAADINLVKIIDSGHNN----------

IR01_SRX802143.125_contig_8432_8 DVFTITQYFLEQRLIEVLKNCNFAAIHANRVKLMPIDIKFVSSLSFGMKNPYTKNIDSLN

.: * * :: :: . .: .:** *: : . **.:. :

human_H2BxH2AxH3 ------------------------------------------------

LCMAC102_QBK86552.1 ------------------------------------------------

IR01_SRX802077.164_contig_92501 -----VKVQVLMES---GEDELTNVSD---------------------

IR01_SRX802202.41_contig_2978_4 ---------YLHDD--WKE----------------------------I

IR01_SRX802963.105_contig_12868_ ---------YLHDD--WKE----------------------------I

IR01_SRX802143.125_contig_8432_8 EICETQEIEFLNDENVYEEECDENVCEEEIEEECEEECEEELDECIGV

**H4**

Homo_H4_NP_001029249.1 MSGRGKGGKGLGKGGAKRHRKVLRDNIQGITKPAIRRLARRGGVKRISGLIYEETRGVLK

IR01_SRX802077.164_contig_92501_ ---------------------------MNITKPSLQRIARQAGIKSMSDECVDIIRTLLH

IR01_SRX802202.41_contig_2978_3 --------------------------MDYITKPSISRLAKRAGIKTISDDCYLIIHESIG

IR01_SRX802963.105_contig_12868_ --------------------------MDYITKPSISRLAKRAGIKTISDDCYLIIHESIG

IR01_SRX802143.125_contig_8432_8 --------------------------MQNITNPSITRLARKAGVKSMSNDCYDSIREIAE

IR01_ERX552261.56_contig_27313_2 --------------------------MDHITKPSITRLARRAGVKSLSEECYPAVRALVA

**:*:: *:*...*:* :* .

Homo_H4_NP_001029249.1 VFLENVIRDAVTYTEHAKRKTVTAMDVVYALKRQGRTL---YGFGG------

IR01_SRX802077.164_contig_92501_ AKTAEICDILILQNN--YTKTIMVDDVYTALKSLNVNLARSDNLGQSVYQLK

IR01_SRX802202.41_contig_2978_3 EEINKIISTALAVN---KTKTLMVEDIQAAFRLNGYNIAKSNDIGSGKY---

IR01_SRX802963.105_contig_12868_ EEINKIISTALAVN---KTKTLMVEDIQAAFRLNGYNIAKSNDIGSGKY---

IR01_SRX802143.125_contig_8432_8 KELEDIVNTILVVNSEHNTKTIMQDDIYDALRLRGHFVARSQELSS------

IR01_ERX552261.56_contig_27313_2 KHLTEVITAAIVVNSEHNTKTLMPEDVYEAFRLRDYNIAQSNDLGTSTCAK-

.: : . **: *: *:. . : :.

**Supplemental Figure S9. Loki’s Castle Histones.**

**H2B-H2A-H3-H4**

Homo_H2BxH2AxH3xH4 MPEPAKSAPAPKKGSKKAVTKAQKKDGKKRKRSRKESYSIYVYKVLKQVHPDTGISSKAM

LCMAC101_QBK85747.1 -MSKTENANQP-------------EEKKASKKKQTNSFQTYIHRLLKSIDPNAQIAVNTR

LCMAC102_QBK86460.1 ----MSSSPTP-------------STQRKSKKKATNSFNVYIHKVLKQVHPDAQISSNTI

..: * . . *.. .:*:. *::.:**.: *:: *: ::

Homo_H2BxH2AxH3xH4 GIMNSFVNDIFERIAGEASRLAHYNKRSTITSREIQTAVRLLLPGELAKHAVSEGTKAVT

LCMAC101_QBK85747.1 GQLDKVADILARGLAEKARTLCIRSKKKTISPSEIYLSMDLLFPRSLAERASEKVNQAIK

LCMAC102_QBK86460.1 GQLDEFIKILAYTLVESARKACIRTNRSTVGKSEISLATNFHFPKILADRATQMLEKACF

* ::.. . : :. .* . .:..*: ** : : :* **..* . :*

Homo_H2BxH2AxH3xH4 KYTSAKXMSGRGKQGGKARAKAKTRSSRAGLQFPVGRVHRLLRK-GNYSERVGAGAPVYL

LCMAC101_QBK85747.1 EYQDAD----KGS-----HTHPVRRETLAGLVFSVALSEKYLREFGASELHVGKMAPIAL

LCMAC102_QBK86460.1 KFTSNI----TKSEKQKERSTPVRRECQAGLLFSVALVEKFIREFGASDLNVSKTSSVAL

:: . . .: . *. *** *.*. . :*: * . .*. :.: *

Homo_H2BxH2AxH3xH4 AAVLEYLTAEILELAGNAARDNKKTRIIPRHLQLAIRNDEELNKLLG--RVTIAQGGVLP

LCMAC101_QBK85747.1 SAILQYVIGEILRFAINCARDLKRVIVKVRHIRLAVGNDPDISQLFQNFKIELMNGGFIP

LCMAC102_QBK86460.1 AAVLESIIREILDLAGNAAHDLHKVIIVIKHVYLAVANDEELDQLRRDLGIEFVGGGVLS

:*:*: : *** :* *.*.* :.. : .*: **: ** ::.:* : : **.:.

Homo_H2BxH2AxH3xH4 NIQAVLLPKKTESHHKAKGKXMARTKQTARKSTGGKAPRKQLATKAARKSAPSTGGVKKP

LCMAC101_QBK85747.1 HIRRELLPSQEK-----------KSAQAARR-------------RKNKKGQPVDS--KRS

LCMAC102_QBK86460.1 GIRAEILPSQEK-----------RTKQAARR-------------RRTKISGTTSS--KKS

*. :**.: : .: *:**. . . . . . *..

Homo_H2BxH2AxH3xH4 HRYRPGTVALREIRRYQKSTELLIRKLPFQRLVREIAQDFKTDLR-------FQSAAIGA

LCMAC101_QBK85747.1 RRLLPGTKAIRDIKHYQKTTECLLQRLPFETSVRGLGKEIRNKDGEEPHLLHFGGGAILT

LCMAC102_QBK86460.1 HKYLPGTKALMDIRRYQKTTTSLLRKLPFNREVRNIAKTINEQVKLSGDVL-FSIGSIDA

.. *** *: :*..***:* *:..***: ** :.: :. . * .:* :

Homo_H2BxH2AxH3xH4 LQEASEAYLVGLFEDTNLCAIHAKRVTIMPKDIQLARRIRGERAXMSGRGKGGKGLGKGG

LCMAC101_QBK85747.1 LQAFVEQRVTKLLNKALDCTLHRSRDSVNAKDVTLAWGMDKDSSWV--------------

LCMAC102_QBK86460.1 LQSFVEQRVTTLFSNALDLAIHSKRDGINGDDVKLAWRLTELS--V--------------

** * :. *:..: ::* .* : .*: **. : :

Homo_H2BxH2AxH3xH4 AKRHRKVLRDNIQGITKPAIRRLARRGGVKRISGLIYEETRGVLKVFLENVIRDAVTYTE

LCMAC101_QBK85747.1 -----LFTENQLEKIGTNGIERLARRGGVKRTSADMYPVVRSFMYSLVSMVLYKCVHIIA

LCMAC102_QBK86460.1 -----RFTPNELKGIGNNGIERLAFRGGVKRKGASIFEIVRQYMYSLVYTILYRVLWYVK

. :::: * . .* *** ****** .. :: .* : :: :: :

Homo_H2BxH2AxH3xH4 HAKRKTVTAMDVVYALKRQGRTL------------------------------YGFGG--

LCMAC101_QBK85747.1 YRKIMTIGVFDLEVAFESFGVNFTIPPHISKAKKSKKSEAKDDSSKDDSSKDDSSKGKSK

LCMAC102_QBK86460.1 YRKVVTIGIKDLRIGFESMGINFTIP---------------------------NAMGKSK

: * *: *: .:: * .: . *

Homo_H2BxH2AxH3xH4 ------------------------------------------------------------

LCMAC101_QBK85747.1 GKSSKDKSKPKGKSSKDKSKSKGKSSKDKSKSKGKSSKDKSKKSQPEETEEKITPKNIPK

LCMAC102_QBK86460.1 SKSHRK------------------------------------------------PTTISE

Homo_H2BxH2AxH3xH4 -----------------------

LCMAC101_QBK85747.1 KASMSKNNKKRNRMKSKKISKTK

LCMAC102_QBK86460.1 K----------------------

**H4-H3**

Homo_H4xH3.3 --M------------SGRGKGGKGL-----GKGGAKRHRKVLRDNIQGITKPAIRRLARR

LCPAC304_QBK91701.1 MSTLPKRKVRPPTYGYGRGKGGKGLVKVGKGLSGAVRHRKVLRDNIQGLTKPAIKRLAHR

LCPAC304_QBK91785.1 MTT------------ATRKKTT-------------DAKTTVTEDPIKGFKDVQIRNLAYR

LCMAC101_QBK85672.1 MAT------------NKKSKNTP-------SQGKVQKRRRILRDNIQGITKPALQRLCHR

. * . : * *:*:.. :..*. *

Homo_H4xH3.3 GGVKRISGLIYEETRGVLKVFLENVIRDAVTYTEHAKRKTVTAMDVVYALKRQGRTLYGF

LCPAC304_QBK91701.1 AGVKKVSGLIYEEIRGVTKVFMEPLIRSCVVIARTEEKKTVKIRHLYGALEIAKTPLAA-

LCPAC304_QBK91785.1 ASAQRIEKDTYGKVREIIGDRIDELLRKILVFTTHTGRKTVTLEDLQGALEAEGTPLMA-

LCMAC101_QBK85672.1 AGIKRISDDMYEILRDSIKTYMERILHKTVIFTEREQRRTVKVKDLEAALNMTGIYLAA-

.. :.:. * * :: ::.. : : ..**. : **: * .

Homo_H4xH3.3 GGXMARTKQTARKSTGGKAPRKQLATKAARKSAPST--------GGVKKPHRYRPGTVAL

LCPAC304_QBK91701.1 AINPTTGSATF-----------FKACEVESRPA-----------GALPKKTRARPGTKAL

LCPAC304_QBK91785.1 GGNTARDIKKSKART-------KKPKQTPKEEGTST--------DLPKKPHRFRPGTVVQ

LCMAC101_QBK85672.1 GLNSNTSKSKT-----------FQSCNSVGKSGPTKKTVKKNEEGGVKKPHRFRPGTKAI

. . . : . . * * **** .

Homo_H4xH3.3 REIRRYQKSTE-LLIRKLPFQRLVRE-IAQDFKTDLRFQSAAIGALQEASEAYLVGLFED

LCPAC304_QBK91701.1 QDIRRQQKSEGCLIFAKLPFERFVRE-IAQDYEIDMQFAADFLLMVQFVCEQYLVGLLFD

LCPAC304_QBK91785.1 KEIKRNQEMSDRLVFKQNRFQYYVRQSTQQQLNVALRFTKEFFKLFQIVIEEYLVCLLDR

LCMAC101_QBK85672.1 RSIRYQQKFSECLAIPKVNFERLVRE-ICQEYKDELRFSEGVFELLQLVVEEYIVVLCKD

..*. *: * : : *: **: *: : :.* : .* . * *:* *

Homo_H4xH3.3 TNLCAIHAKRVTIMPKDIQLARRIRGERA

LCPAC304_QBK91701.1 ANSAAIHDGRQTIFPKDIQLARMIRGERA

LCPAC304_QBK91785.1 AIKASAHAGRKSLSEKDLQFVYSL-----

LCMAC101_QBK85672.1 AYSCAIHAERTTIKAKDINLVRKLRR---

: .: * * :: **:::. :

**H2B-H2A**

Homo_H2BxH2A ---MPEPAKSAPAPKKGSKKAVTKAQKKDGKKRKRSRKESYSIYVYKVLKQVHPDTGISS

LCPAC001_QBK89657.1 ---------MNFILRLIDKKSLSILYLKMSKNSQNSLFPNYTLYISKILKLVICSGSMKK

LCMAC201_QBK87604.1 ----------------------------MSRQKTARTYDKLHTFIYHLLKHYDPKANMTA

LCMAC202_QBK88134.1 -------------------------MNSKVPKKKVRSYDNLHTFIYRLLKHYDPKANMTA

LCPAC304_QBK91784.1 MATKAKAKTTKPKTVSDRGSGVSASKNVVDGPKKKKNYDTFKKFIQDLLKK-QRGGGISG

LCMAC101_QBK85671.1 ----------------------------------MGLDANFEIYIRRVLKQVHPDAGISG

LCPAC304_QBK91702.1 --------------------------------MPKSKTPRYATFIYKVLKQVHPDTGING

:: :** .:.

Homo_H2BxH2A KAMGIMNSFVNDIFERIAGEASRLAHY--NKRSTITSREIQTAVRLLLP-----------

LCPAC001_QBK89657.1 ESNEQMNKIVDIIATNIVKGAYESTLS--LKKKILSEEELRTSLKIIFS-----------

LCMAC201_QBK87604.1 ECKDILNHLIRDLSLRYITASVELCRY--AKKVTIDFNAIETLTRIWIV-----------

LCMAC202_QBK88134.1 ECKDILNHLIRDLSLHYITACVELCRY--AKKVTIDSNAIETLTNIWIT-----------

LCPAC304_QBK91784.1 GALDVTDRYVKIILEMIIRNADLLLAR--SKKKTLSEKEIESAVRLTIPPPHRSEFSSEP

LCMAC101_QBK85671.1 SALASLNNLVKITIQKIMVAINRILLA--TGKKTINSRDVQDAIRLILP-----------

LCPAC304_QBK91702.1 DALAEMNRLILTILFEIIRVADAINRSFNNPKITFGTRTVQGAIKLALP-----------

. : : . : : .: :

Homo_H2BxH2A -----GELAKHAVSEGTKAVTKYTSAKXMSGRGKQGGKARA-------KAKTRSSRAGLQ

LCPAC001_QBK89657.1 -----KETATSIIKFSETILVKLDDFSAENKI----------------KRISLPKTLEVF

LCMAC201_QBK87604.1 -------TPDSILDFAHDTWTMYSQNTT--------------------IGIKKERRAGLF

LCMAC202_QBK88134.1 -------NPDYLIDFAHDTWDVYSQNTT--------------------KGIKKSCRAGLF

LCPAC304_QBK91784.1 GSEPPRNLAVEANKRGAEAVRKFMSAQNNKVKGQ--------------GRSTKSKKADLV

LCMAC101_QBK85671.1 -----GEVRKHAISEGTKAVTKYNSSKLNASAGD--------------KPTMKSTRAGLS

LCPAC304_QBK91702.1 -----GELSRHAISEGCNAVTKYNSSTAAAKEDKESRPKRSFKDSRTGGKKTKAFRSGLM

. . . :

Homo_H2BxH2A FPVGRVHRLLR--KGNYSERVGAGAPVYLAAVLEYLTAEILELAGNAARDNKKTRIIPRH

LCPAC001_QBK89657.1 YPIKMLKNILL-KLGKKNIKMSLNADLYFITFMFYITHLILELSYNEASIWKRTRLGPRD

LCMAC201_QBK87604.1 LPPARFKELFREYRGANQ-KIGEPAYIFLTAIIEAIFGLIIESAIKLVQGEKKITVTGLH

LCMAC202_QBK88134.1 LPPARFRELFREYRGANQ-KIGEPAYIFLTAVIEAIFGLIIERAVHLARDEHKITVSGLH

LCPAC304_QBK91784.1 FPVSRVKDRISESSSSDGLRVGEKSIVYLTAVLQYLTEELLQMAGEIADSKKHVRITPRD

LCMAC101_QBK85671.1 FSVPRTQKLMM-RMSSVQ-RKTGDAAVYITAVCEYLMAEVMELAGNAAKDQKRVRVTPRH

LCPAC304_QBK91702.1 FPPTRVGDDTRERMASFD-RLDSRTPVYLAAVLEYLTAEILELAGNSARDNHLVRIRARD

. . . : ::: :. : ::: : . : :

Homo_H2BxH2A LQLAIRNDE--ELNKLLGRVTIAQGGVLPNIQAVLLPKKTESHHKAKGK-------------------------------

LCPAC001_QBK89657.1 ILIGINGDE--EFRALFNKLDIYID--------------TKHYKYPYKG-------------------------------

LCMAC201_QBK87604.1 VYRAINSSDFAYLYPLF-RNSFIAG--FGYIGDIQLINAQKRYLISKYH-------------------------------

LCMAC202_QBK88134.1 IYNAINSKDFSHLYPLW-NNSFIAG--FGYIGNTLLQNAQERYIERKQNDREVTPDTAINKIKIFNYKLNL---------

LCPAC304_QBK91784.1 LKLAIENDA--SLVKLT-ENVYIPGGV---------------RVRPKKR-------------------------------

LCMAC101_QBK85671.1 IKIAIYNDE--ELCILY-KDTVFAGGVLPDIDPKVLEKKGKKKAALKNTKNKNTKATKKATKKKSATKSAKKGKPGKKTN

LCPAC304_QBK91702.1 IYLATQADE--ELTKLL-KGMIVPGGVVPFIHQCLLP-----KPKPKTA-------------------------------

: . . : * .
